# Supplementary material for: Phage proteins target and co-opt host ribosomes immediately upon infection
Source: Nat Microbiol. 2024 Mar 4;9(3):787–800. doi: 10.1038/s41564-024-01616-x (PMC10914614; doi:10.1038/s41564-024-01616-x)
Supplement: Supplementary file 1 — Reporting Summary [file 41564_2024_1616_MOESM1_ESM.pdf]

nature portfolio

Corresponding author(s): Prof. Dr. Jörg Vogel

Last updated by author(s): Jan 15, 2024

Reporting Summary

Nature Portfolio wishes to improve the reproducibility of the work that we publish. This form provides structure for consistency and transparency in reporting. For further information on Nature Portfolio policies, see our Editorial Policies and the Editorial Policy Checklist.

Statistics

For all statistical analyses, confirm that the following items are present in the figure legend, table legend, main text, or Methods section.

n/a	Confirmed	
☐	☒	The exact sample size (*n*) for each experimental group/condition, given as a discrete number and unit of measurement
☐	☒	A statement on whether measurements were taken from distinct samples or whether the same sample was measured repeatedly
☐	☒	The statistical test(s) used AND whether they are one- or two-sided<br>*Only common tests should be described solely by name; describe more complex techniques in the Methods section.*
☒	☐	A description of all covariates tested
☒	☐	A description of any assumptions or corrections, such as tests of normality and adjustment for multiple comparisons
☐	☒	A full description of the statistical parameters including central tendency (e.g. means) or other basic estimates (e.g. regression coefficient) AND variation (e.g. standard deviation) or associated estimates of uncertainty (e.g. confidence intervals)
☐	☒	For null hypothesis testing, the test statistic (e.g. *F*, *t*, *r*) with confidence intervals, effect sizes, degrees of freedom and *P* value noted<br>*Give P values as exact values whenever suitable.*
☒	☐	For Bayesian analysis, information on the choice of priors and Markov chain Monte Carlo settings
☒	☐	For hierarchical and complex designs, identification of the appropriate level for tests and full reporting of outcomes
☐	☒	Estimates of effect sizes (e.g. Cohen's *d*, Pearson's *r*), indicating how they were calculated

Our web collection on statistics for biologists contains articles on many of the points above.

Software and code

Policy information about availability of computer code

Data collection	no software was used for data collection
Data analysis	cutadapt (4.1), READemption (1.0.1), cLinker (0.0.24), MotionCor2 (1.4.2), CryoSPARC (3.3.2), AlphaFold2 (2), Colabfold - AlphaFold2_mmseqs (1.3.0), ChimeraX (1.4), Phenix (1.20.1-4487), WinCoot (0.9.8.1), DelPhi (8.5.0), MaxQuant (v1.6.17.0), Porechop (v0.2.3), Flye (v2.9.1), minimap2 (v2.17), IGV (v2.16.1), Grad-seq browser (1.0)

For manuscripts utilizing custom algorithms or software that are central to the research but not yet described in published literature, software must be made available to editors and reviewers. We strongly encourage code deposition in a community repository (e.g. GitHub). See the Nature Portfolio guidelines for submitting code & software for further information.

Data

Policy information about availability of data

All manuscripts must include a data availability statement. This statement should provide the following information, where applicable:

- Accession codes, unique identifiers, or web links for publicly available datasets
- A description of any restrictions on data availability
- For clinical datasets or third party data, please ensure that the statement adheres to our policy

MS data are deposited at the ProteomeXchange consortium via the PRIDE partner repository (Perez-Riverol et al., 2022) with the data set identifier PXD038771 for ΦKZ infected cells. Raw data after MaxQuant and sequencing analysis are listed in Suppl. Table 1. Sedimentation data can also be viewed in a user-friendly browser

at www.helmholtz-hiri.de/en/datasets/gradseqphage. Raw sequencing data and coverage files are accessible at Gene Expression Omnibus (Barrett et al., 2012) with the accession number GSE223979, the analysed data are listed in Suppl. Table 2. Cryo-EM density maps of 70S–tRNA(P)–ΦKZ014, 70S–ΦKZ014 (focused), 70S–tRNA(E)–ΦKZ014 were deposited at EMDB under accession number EMD-16566. The final model of 70S–tRNA(P)–ΦKZ014 was deposited at RCSB-PDB 8CD1. Strains, oligonucleotides, plasmids, antibodies, software are listed in Suppl. Table 4.

Research involving human participants, their data, or biological material

Policy information about studies with human participants or human data. See also policy information about sex, gender (identity/presentation), and sexual orientation and race, ethnicity and racism.

Reporting on sex and gender	n/a
Reporting on race, ethnicity, or other socially relevant groupings	n/a
Population characteristics	n/a
Recruitment	n/a
Ethics oversight	n/a

Note that full information on the approval of the study protocol must also be provided in the manuscript.

Field-specific reporting

Please select the one below that is the best fit for your research. If you are not sure, read the appropriate sections before making your selection.

☒ Life sciences ☐ Behavioural & social sciences ☐ Ecological, evolutionary & environmental sciences

For a reference copy of the document with all sections, see nature.com/documents/nr-reporting-summary-flat.pdf

Life sciences study design

All studies must disclose on these points even when the disclosure is negative.

Sample size	No statistical method was used to predetermine sample size. Bacterial assays were completed in two independent experiments and the error accounts were reported. All attempts to replicate data were successful. Grad-seq data are a representative example of two independent experiments.
Data exclusions	We did not observe outliers in our data that needed to be excluded.
Replication	Bacterial assays were completed in two independent experiments and the error accounts were reported. All attempts to replicate data were successful. Grad-seq data are a representative example of two independent experiments. Representative experiments were repeated two (Figs. 3c,d(ΦKZ105/-206), 4b-e, 5e, 6c,d,f, Ext. Data Figs. 1b, 2, 3a, 5a-c), three (Figs. 4f, 5a, 6a,e), four times (Figs. 3c,d(ΦKZ014), 4g,h, 6b,g) with similar results. We added a "Statistics and Reproducibility" section in the methods part.
Randomization	No data were excluded from the analyses and the experiments were not randomized.
Blinding	This study does not involve procedures that require blinding. The investigators were not blinded to allocation during experiments and outcome assessment.

Reporting for specific materials, systems and methods

We require information from authors about some types of materials, experimental systems and methods used in many studies. Here, indicate whether each material, system or method listed is relevant to your study. If you are not sure if a list item applies to your research, read the appropriate section before selecting a response.

Materials & experimental systems

n/a	Involved in the study
☐	☒ Antibodies
☒	☐ Eukaryotic cell lines
☒	☐ Palaeontology and archaeology
☒	☐ Animals and other organisms
☒	☐ Clinical data
☒	☐ Dual use research of concern
☒	☐ Plants

Methods

n/a	Involved in the study
☒	☐ ChIP-seq
☒	☐ Flow cytometry
☒	☐ MRI-based neuroimaging

Antibodies

Antibodies used	anti-FLAG (mouse, 1:3k, Sigma, F1804), anti-His (mouse, 1:3k, Sigma, A7058), anti-GFP (mouse, 1:1k, Sigma, 11814460001 (Roche)), anti-mouse-HRP (goat, 1:10k, Thermo Scientific, 31430), anti-rabbit-HRP (goat, 1:10k, Thermo Scientific, 31460), anti-ΦKZ014.1 (1660, rabbit, 1:10k, Eurogentec, produced against peptide EQYGESDDTSDESSY, Ext. Data 4d).
Validation	For anti-FLAG, anti-His, anti-GFP, anti-mouse and anti-rabbit, see the product information sheets of the manufacturers (Sigma, Thermo Scientific), these were also validated in previous publications from the Vogel lab with various ectopically expressed targets (e.g. Gerovac et al. 2020 RNA). anti-ΦKZ014 antibodies were produced by Eurogentec and validated in Fig. 4c,g,h and Extended Data 2 with uninfected vs infected PAO1 cells by PHIKZ phage, PHIKZ014 deleted strains, and with purified PHIKZ014 protein.
