# Supplementary figures and images for: Phage proteins target and co-opt host ribosomes immediately upon infection
Source: Nat Microbiol. 2024 Mar 4;9(3):787–800. doi: 10.1038/s41564-024-01616-x (PMC10914614; doi:10.1038/s41564-024-01616-x)

Fig. 3c

ΦKZ014

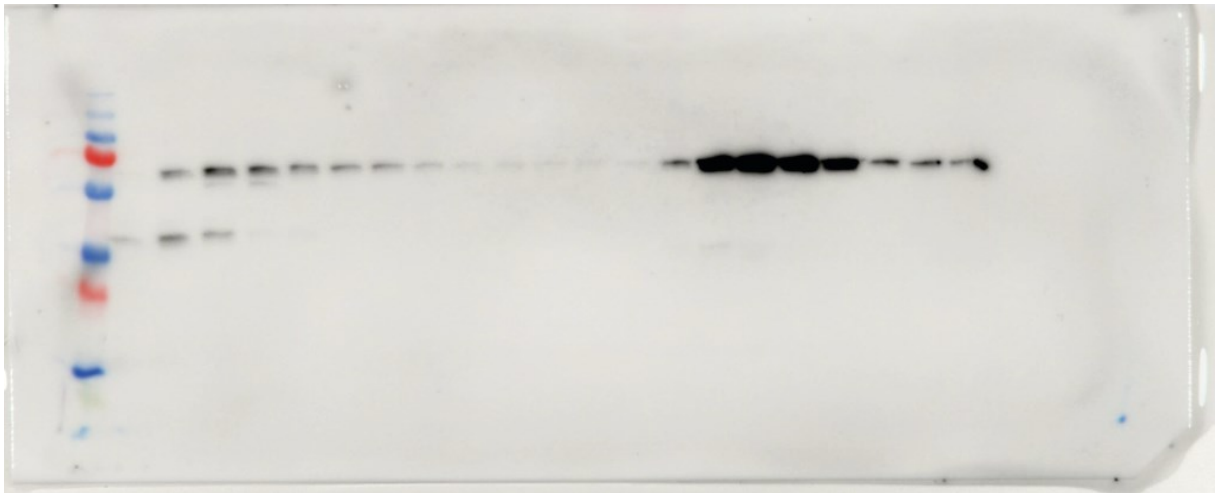

ΦKZ105

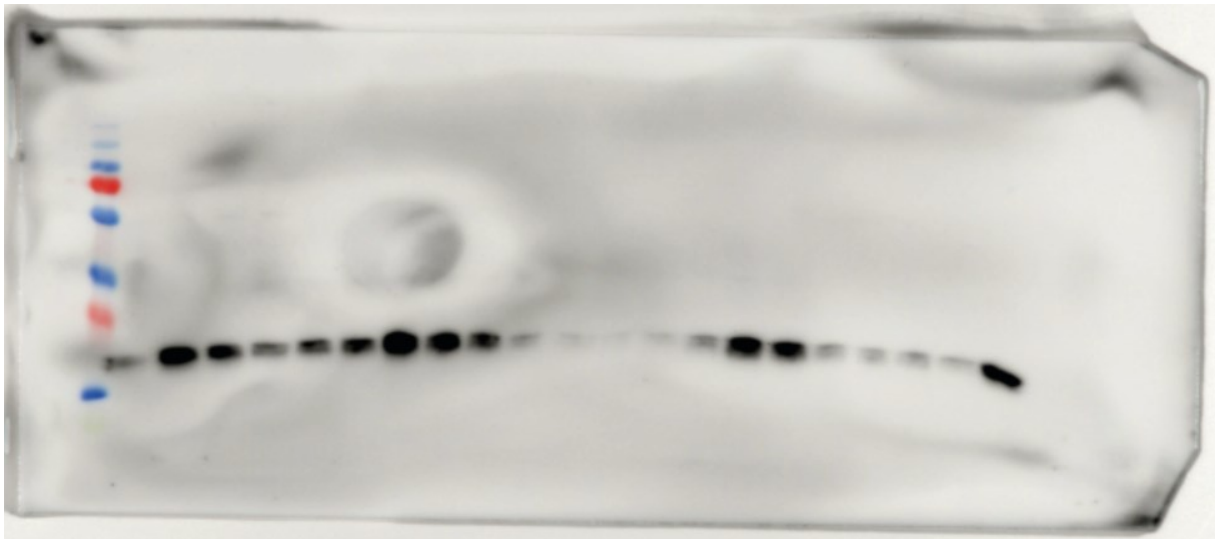

ΦKZ206-GFP

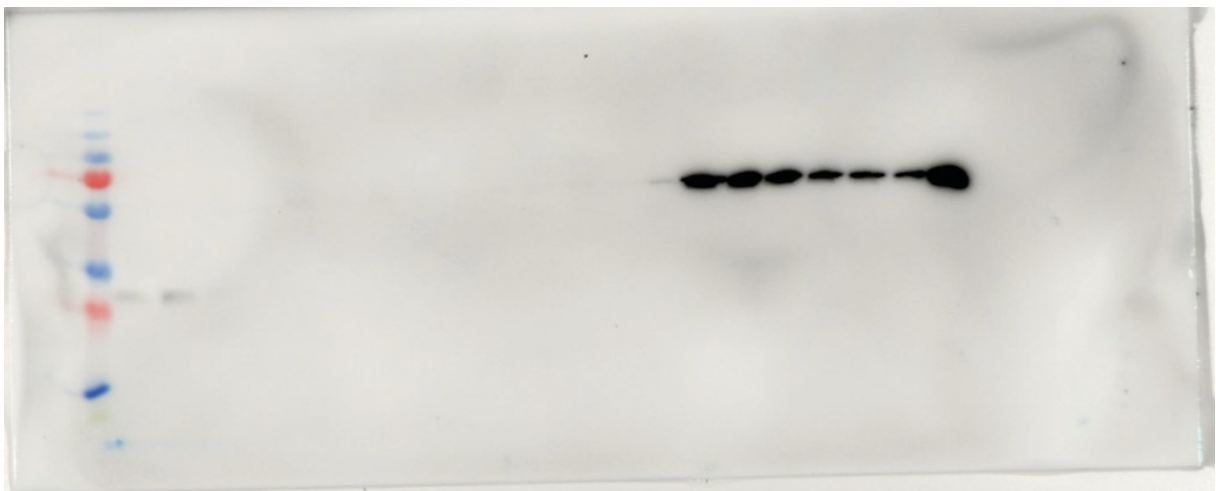

Fig. 3d

$\Phi$ KZ014

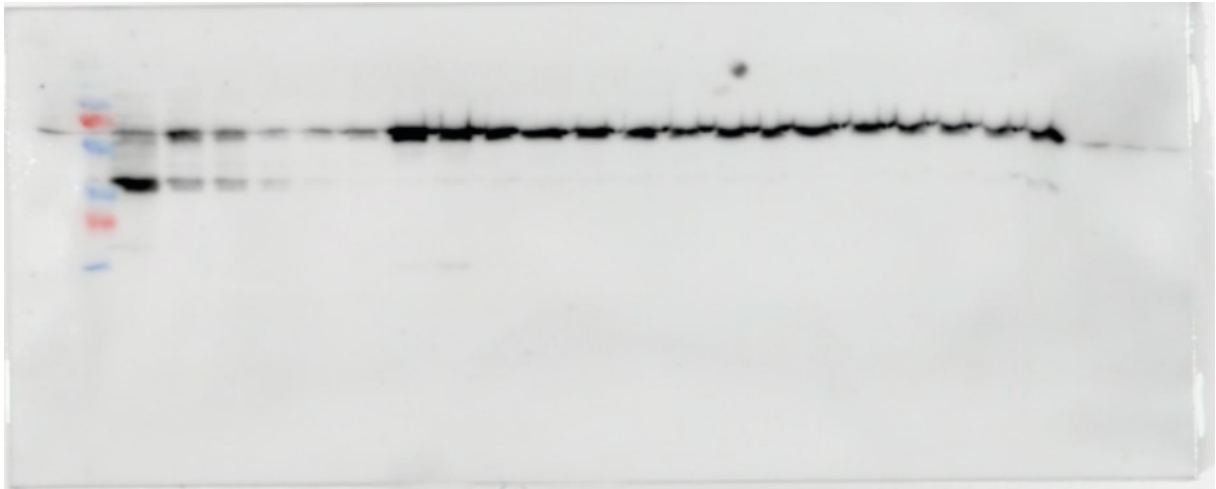

$\Phi$ KZ105

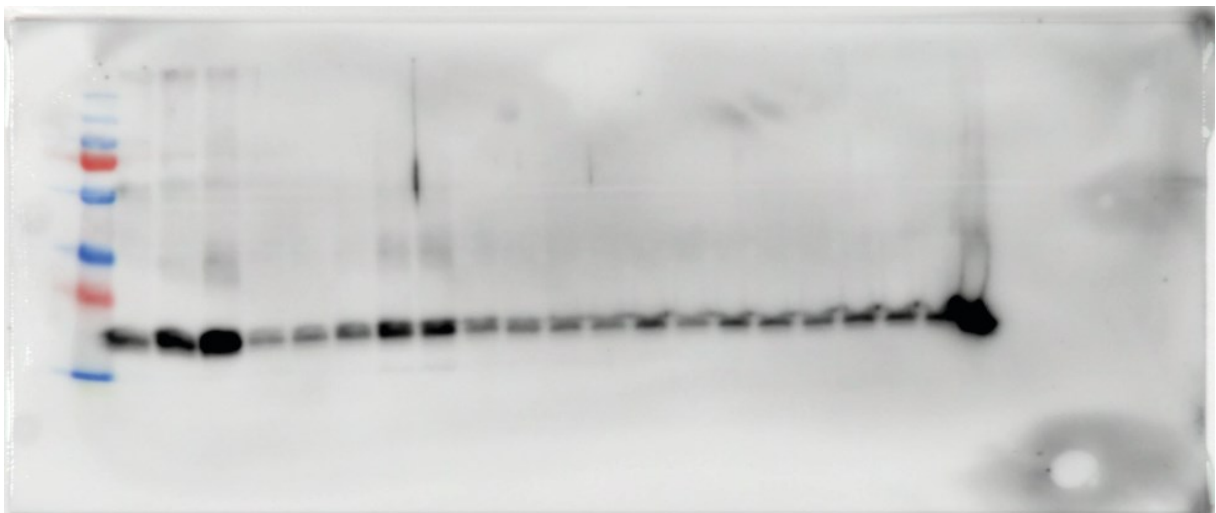

$\Phi$ KZ206-GFP

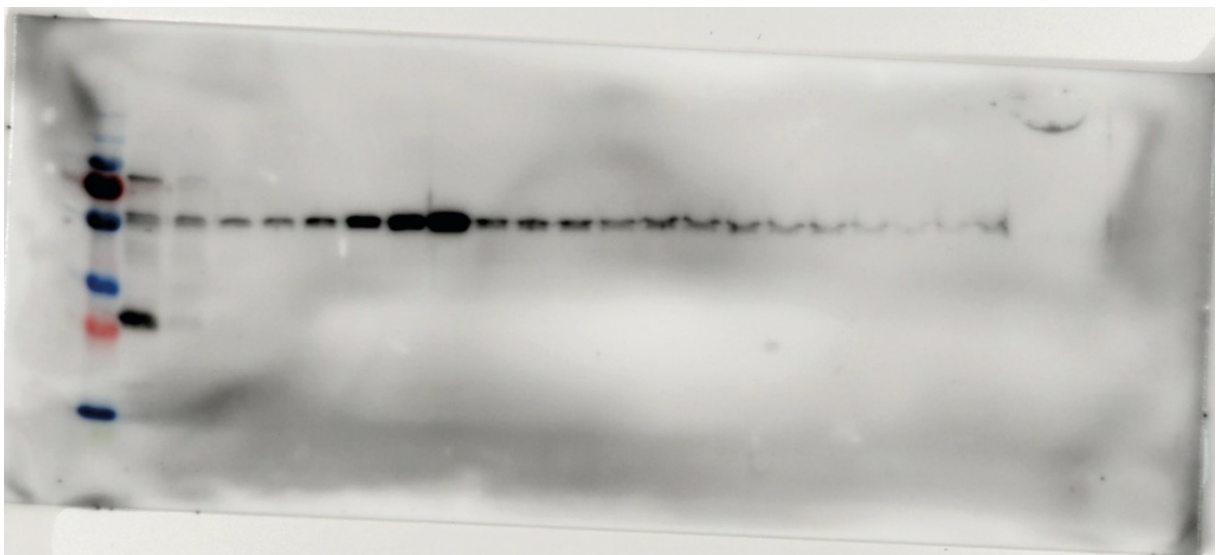

Supplement: Supplementary file 6 — Unprocessed western blots. [file 41564_2024_1616_MOESM6_ESM.pdf]

**Fig. 5a**

anti-His

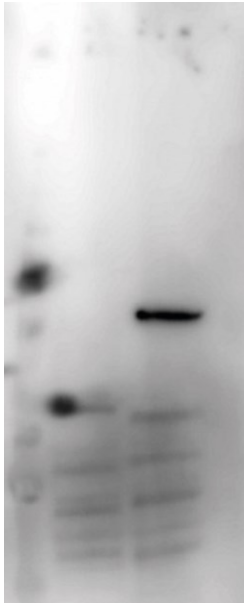

Coomassie

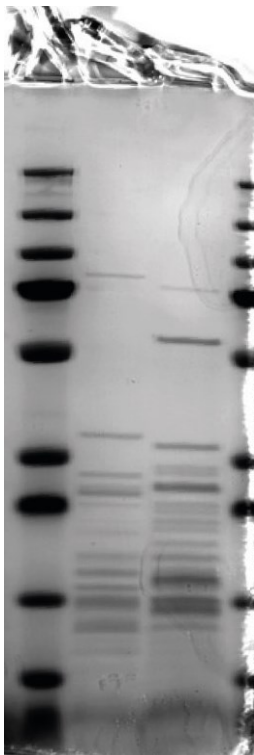

Fig. 5e

$\Phi$ KZ014(R10A)

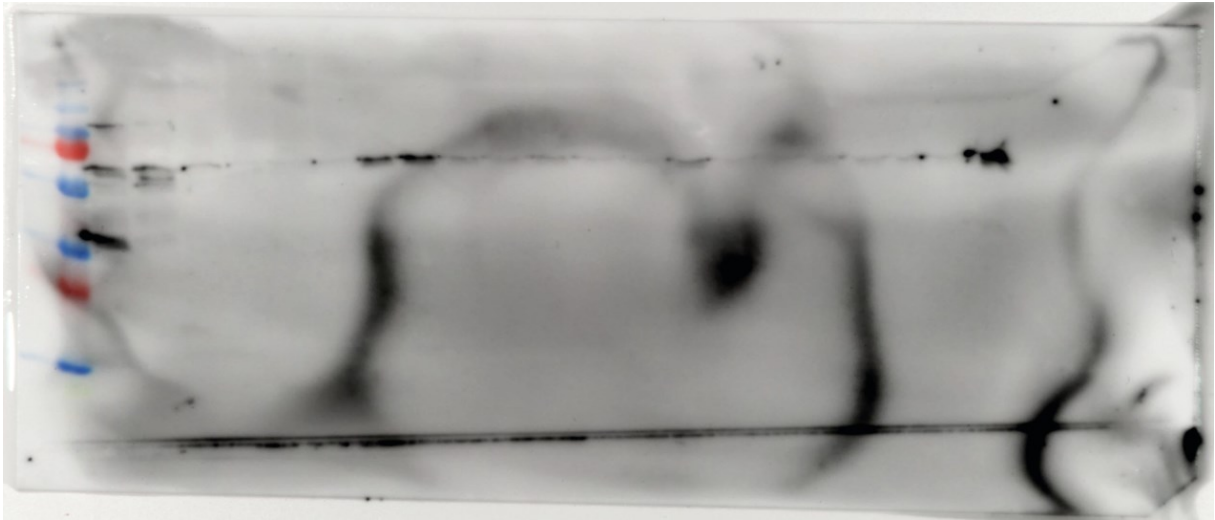

$\Phi$ KZ014(K15A)

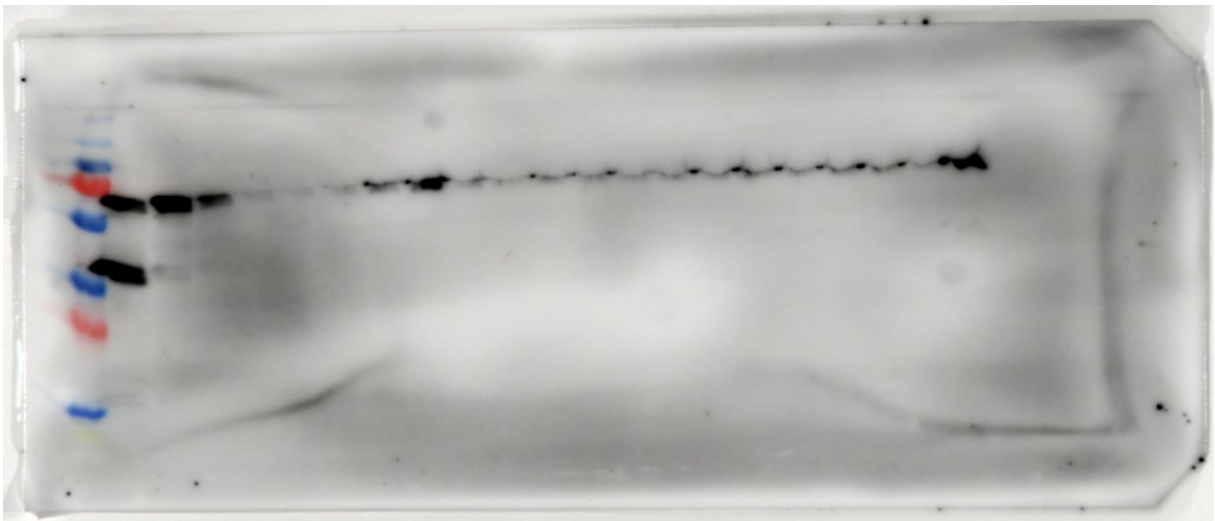

$\Phi$ KZ014(R23A)

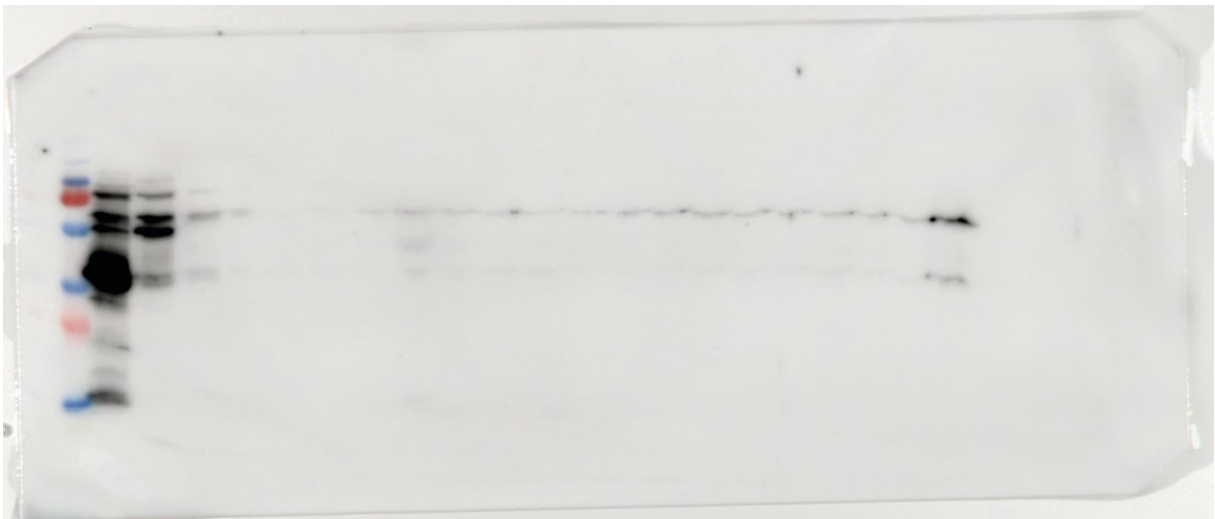

ΦKZ014(K15A,R23A)

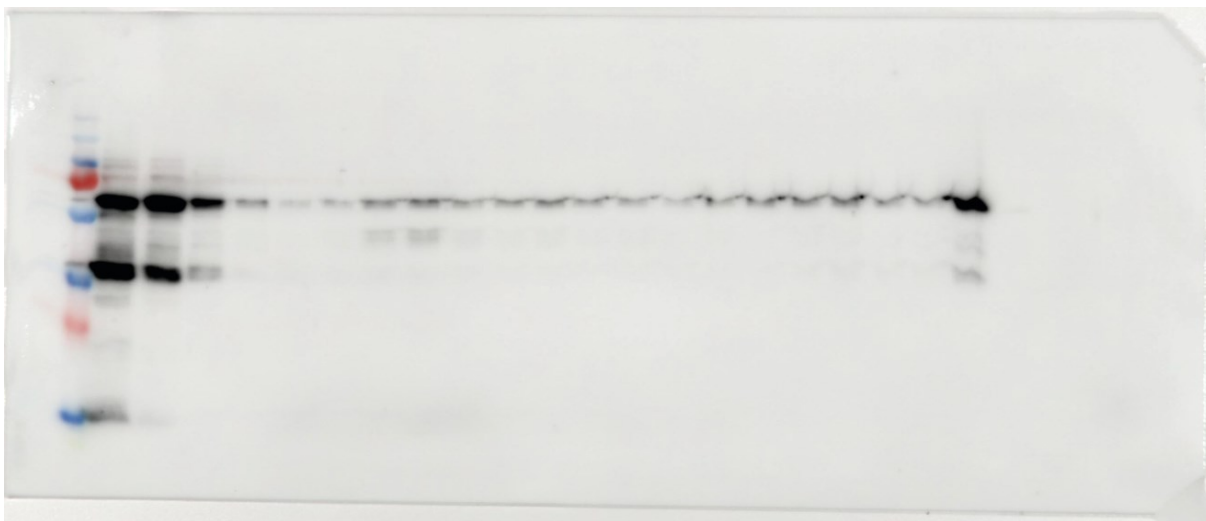

ΦKZ014(K152A)

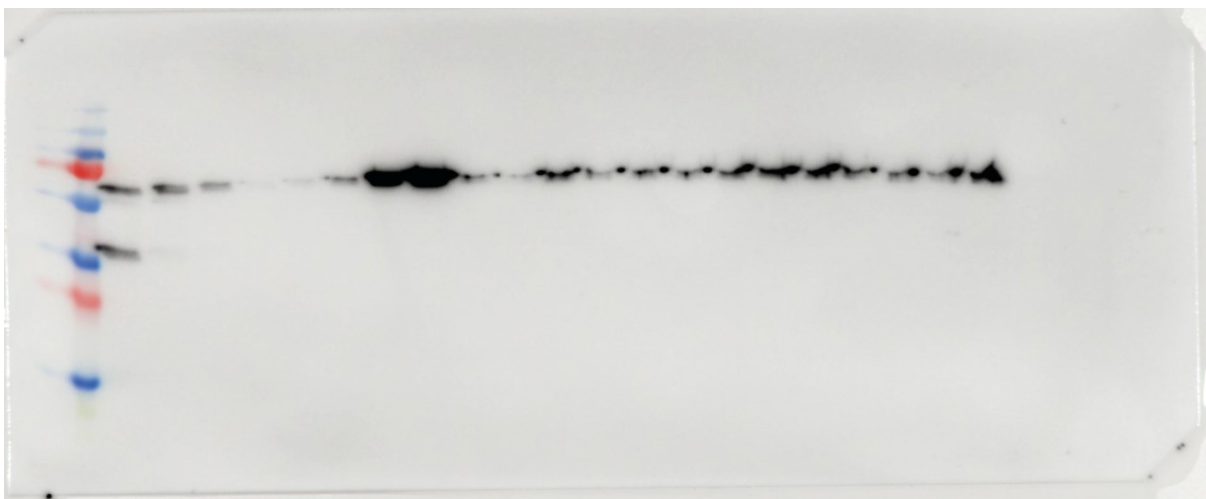

ΦKZ014(R262A)

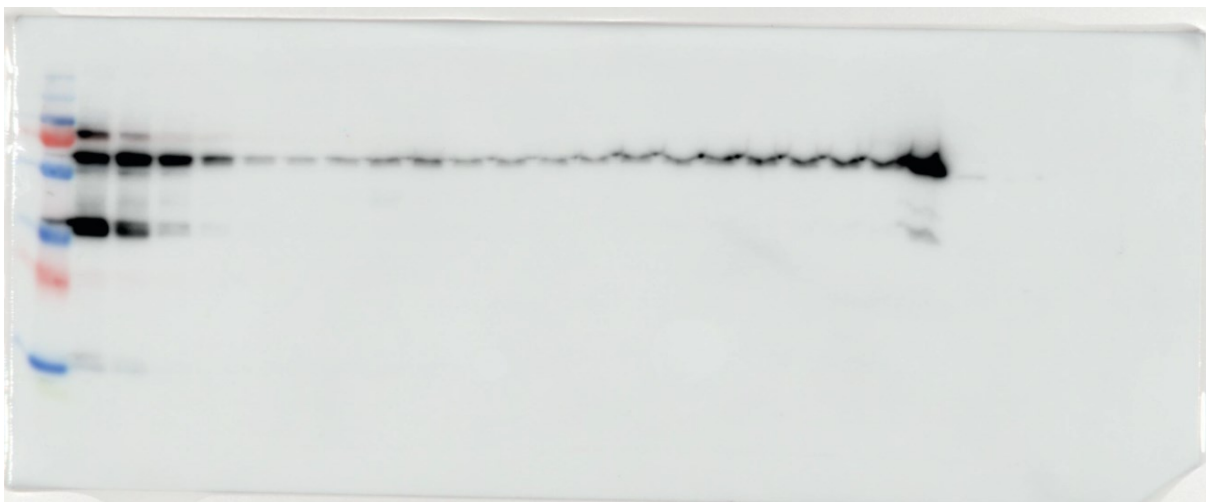

Supplement: Supplementary file 10 — Unprocessed western blots. [file 41564_2024_1616_MOESM10_ESM.pdf]

Fig. 6a

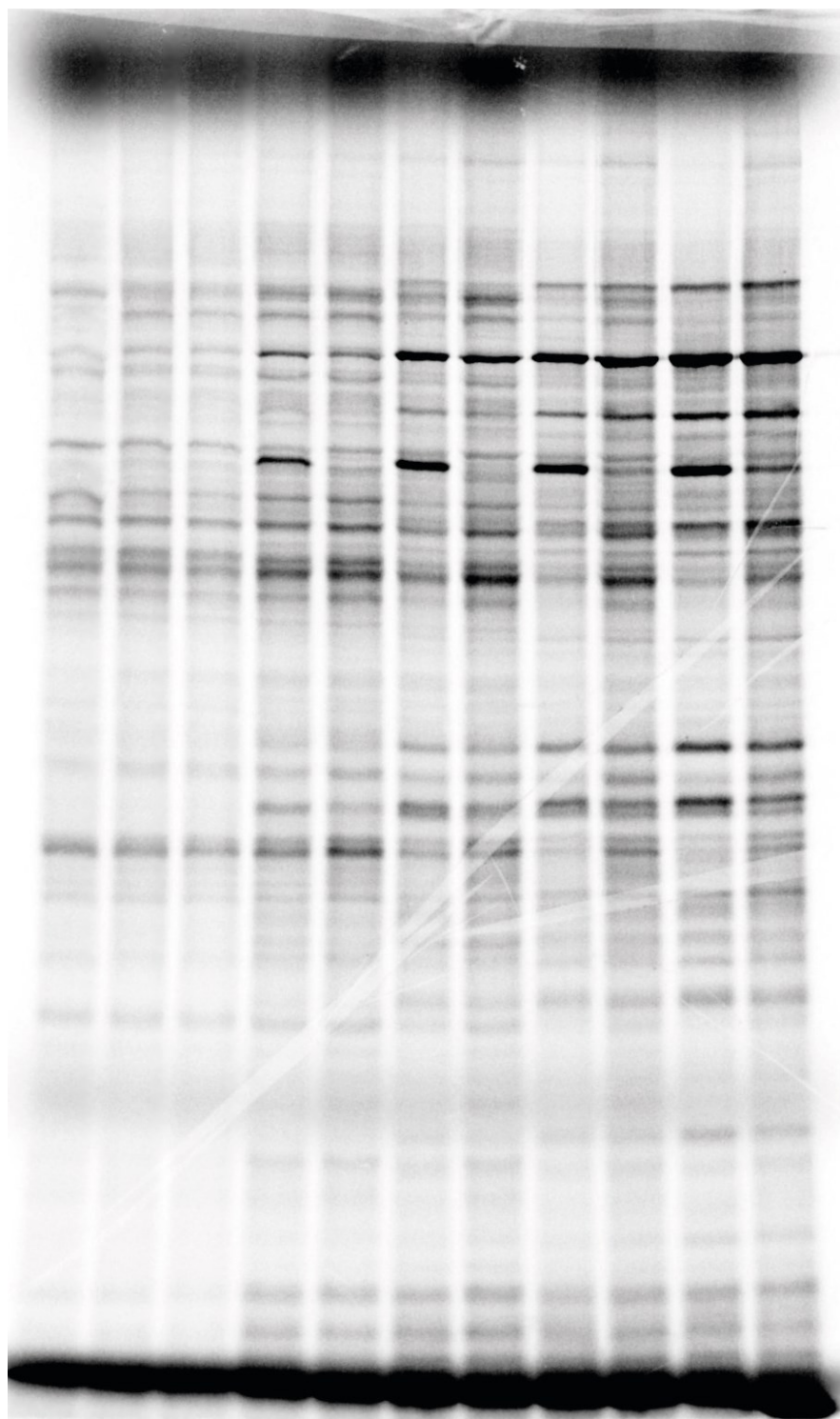

**Fig. 6b**

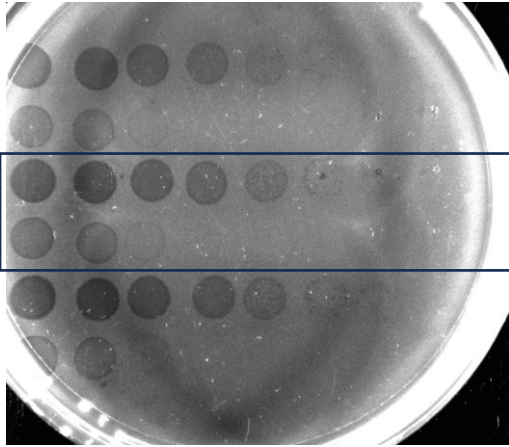

**Fig. 6d**

+ pempty

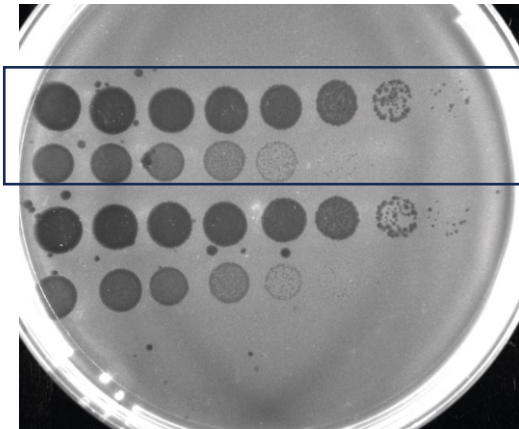

+pPHIKZ014

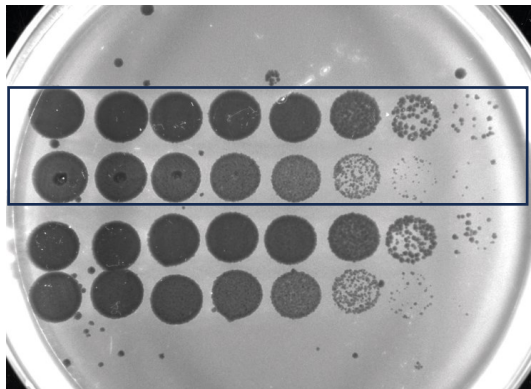

**Fig. 6e**

n.i.

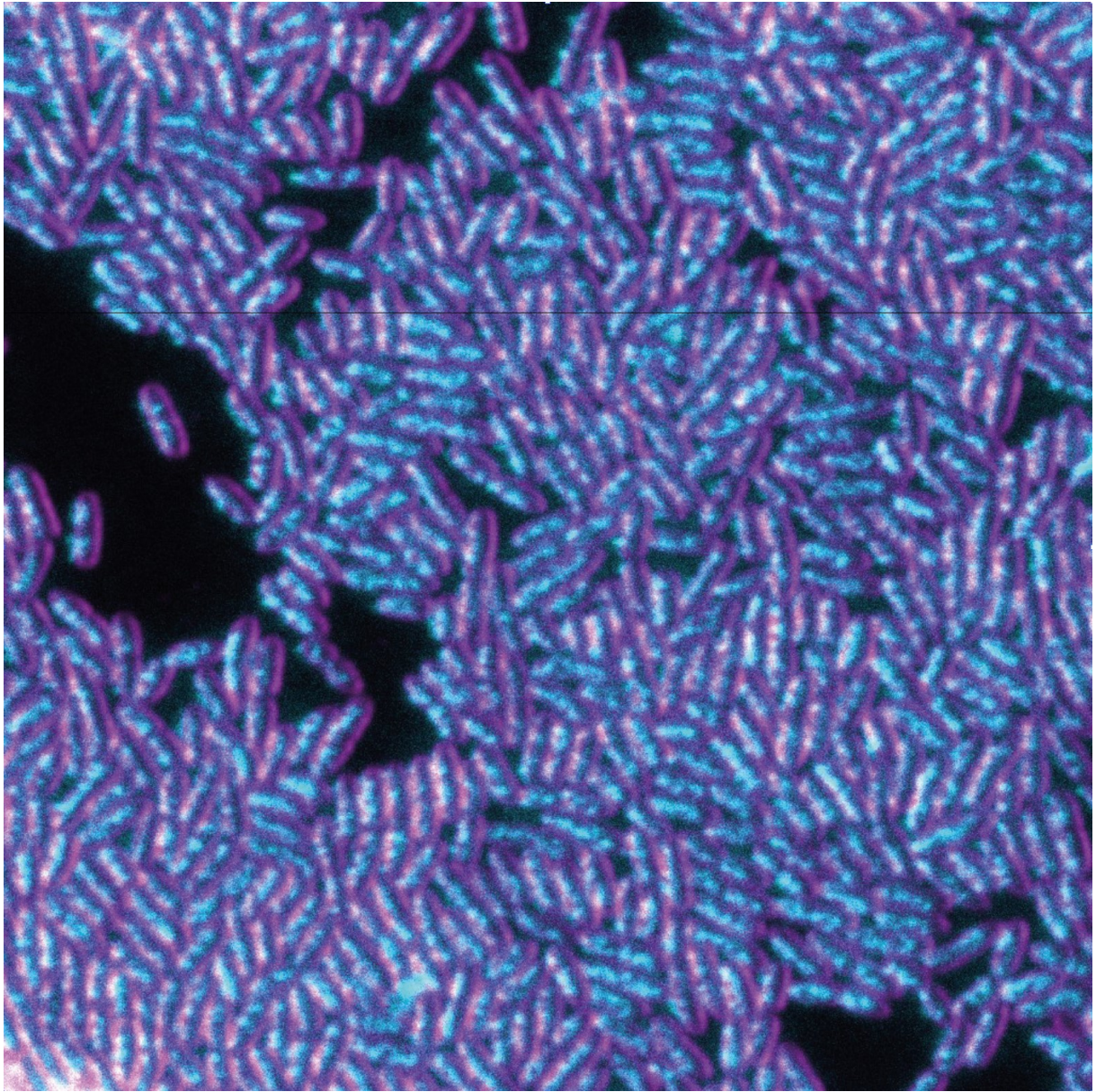

2.5 min wt

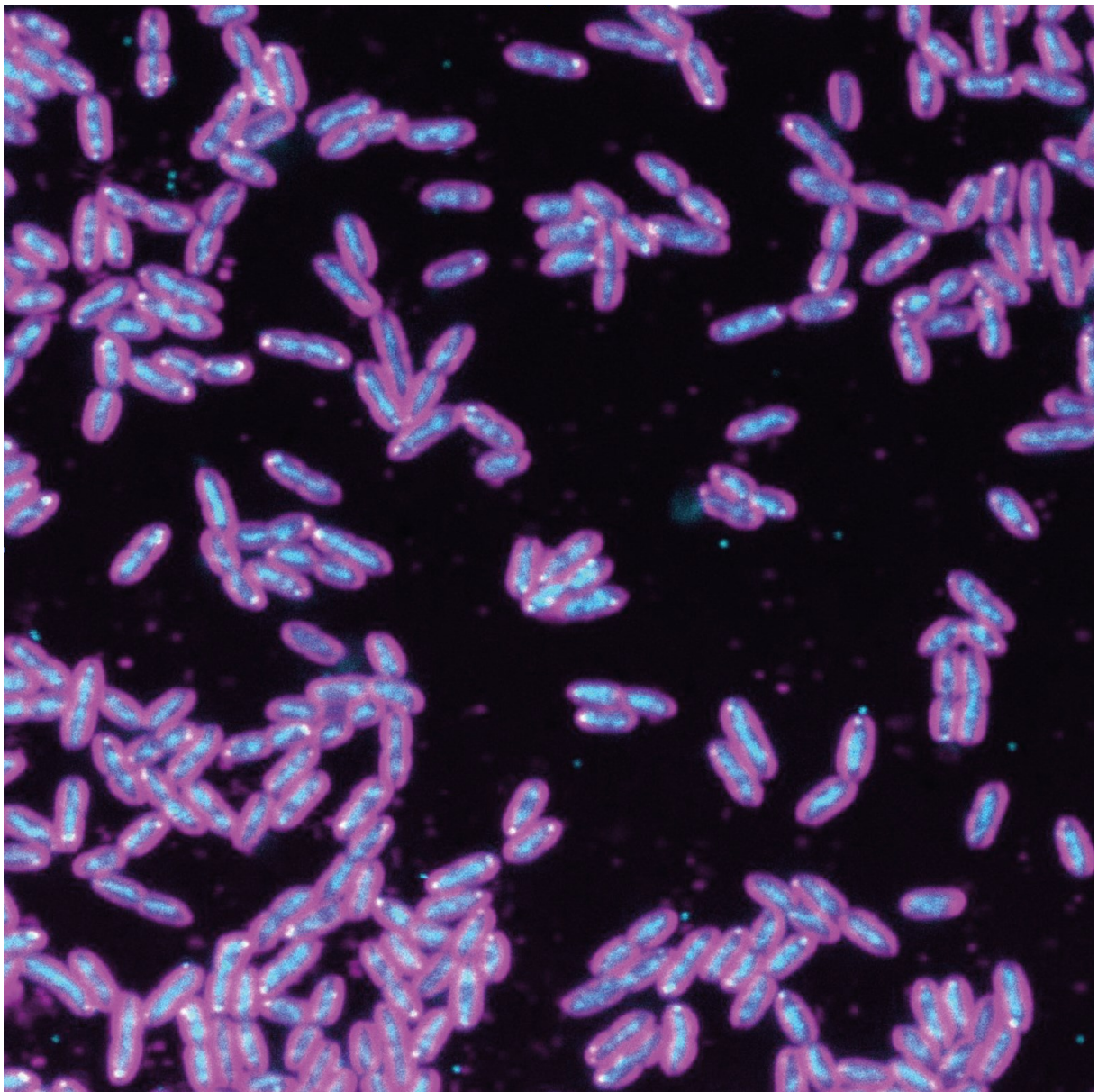

2.5 min  $\Delta\Phi KZ014$

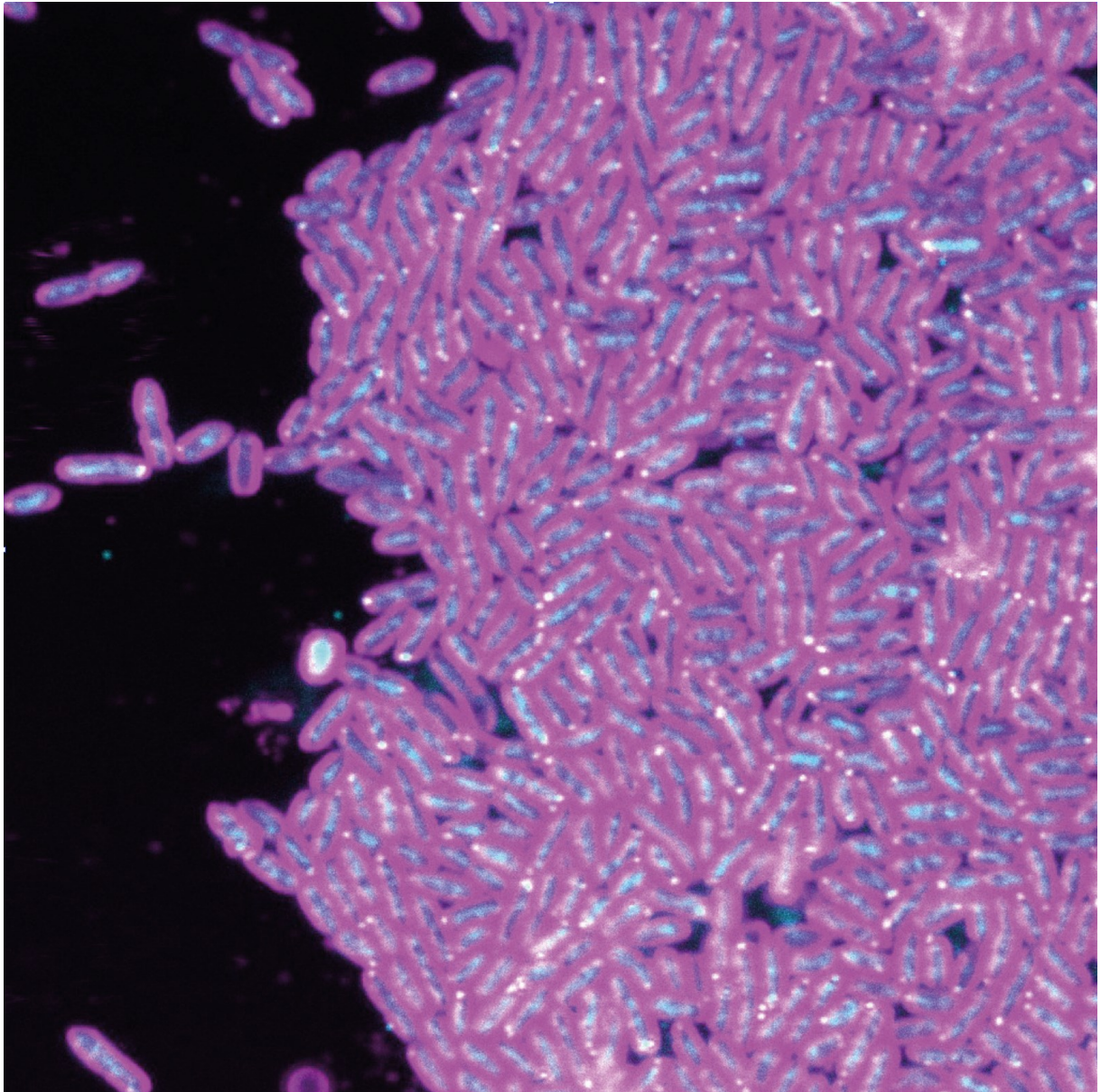

35 min wt

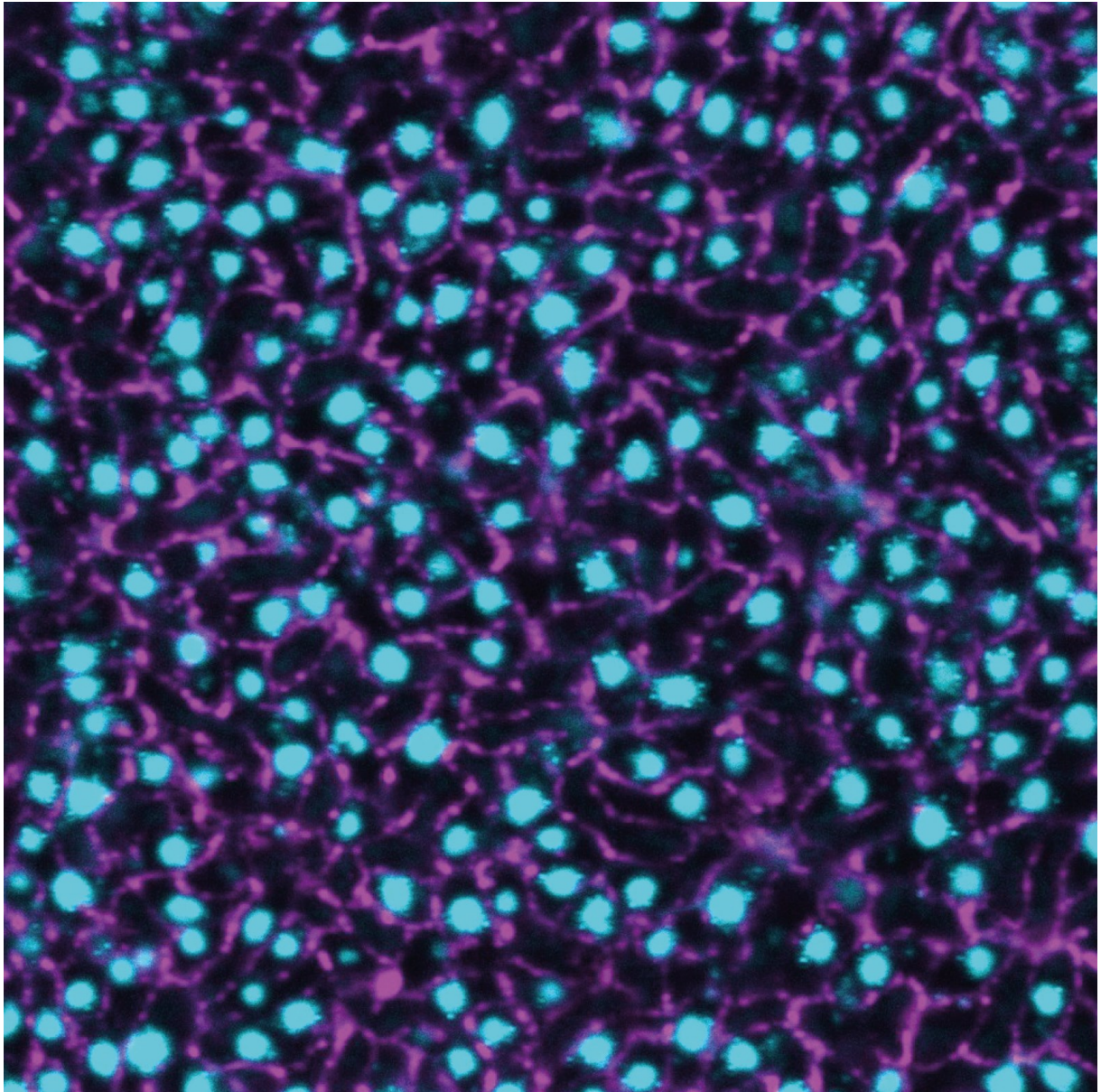

35 min  $\Delta\Phi\text{KZ014}$

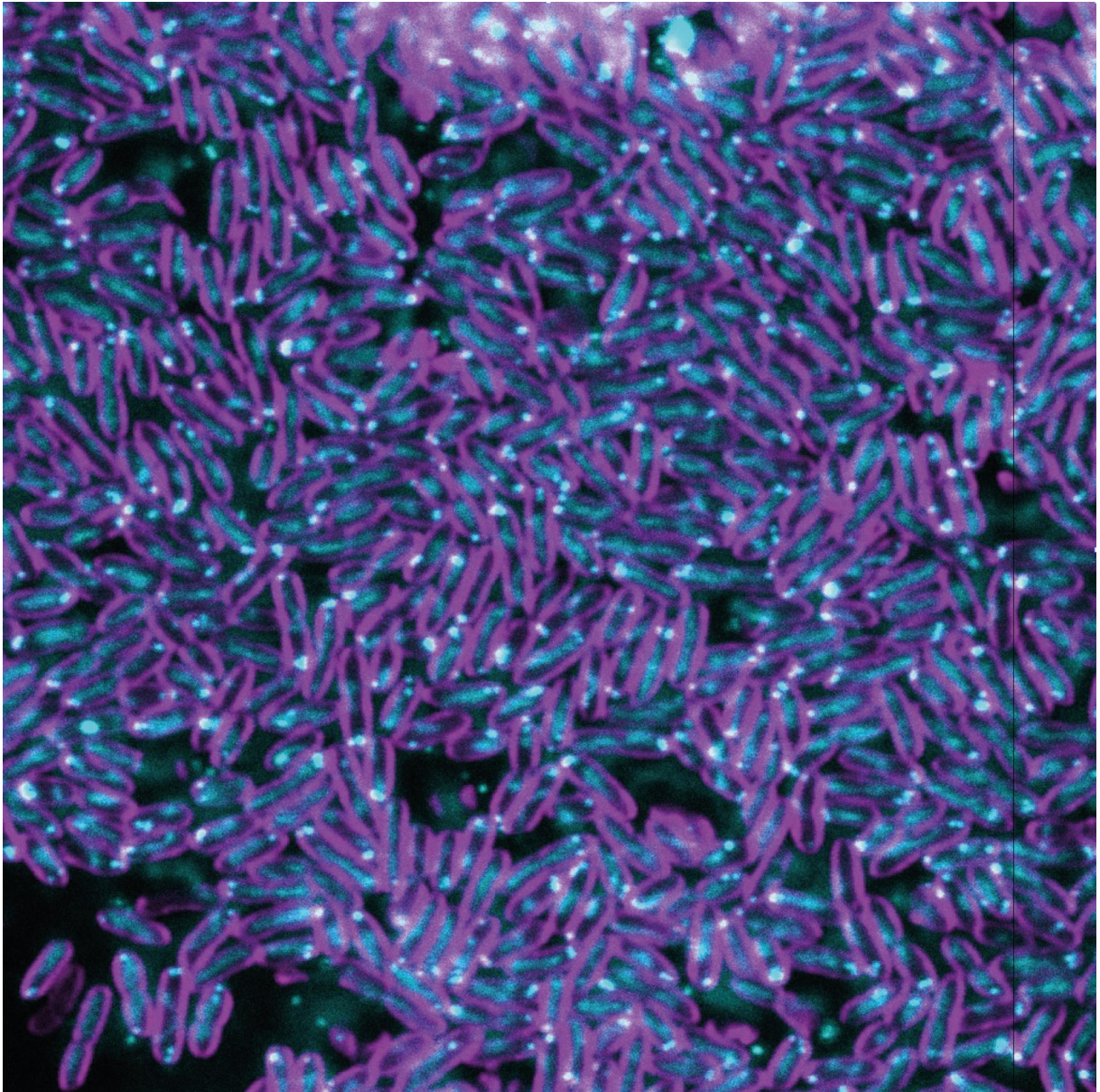

Fig. 6f

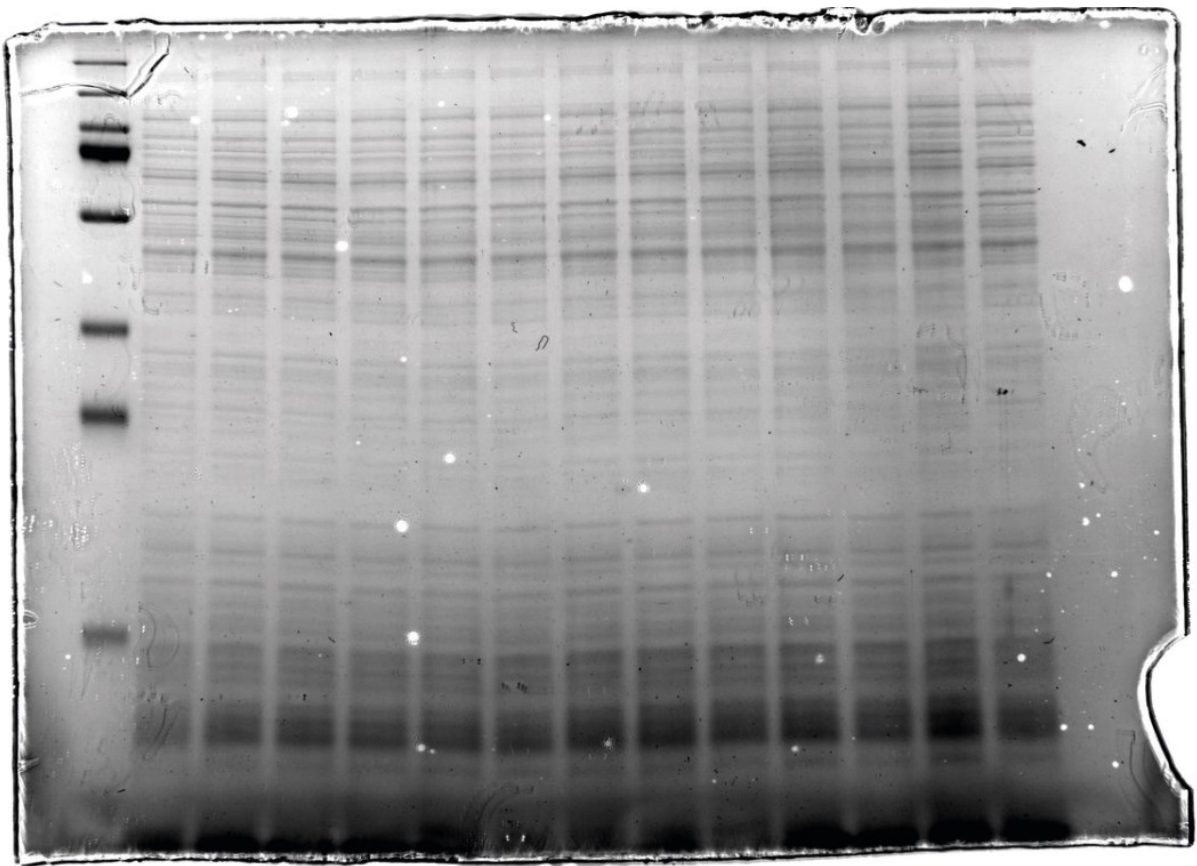

Fig. 6g

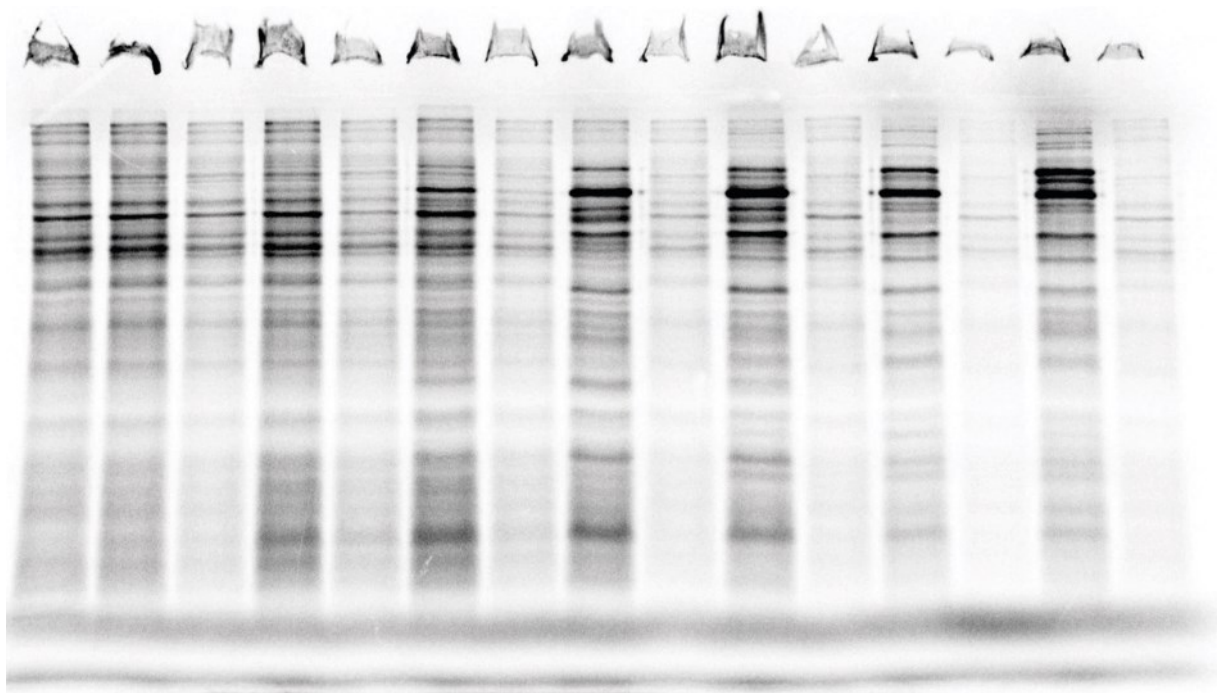

Supplement: Supplementary file 11 — Unprocessed western blots. [file 41564_2024_1616_MOESM11_ESM.pdf]

Ext. Data Fig. 1b

Coomassie

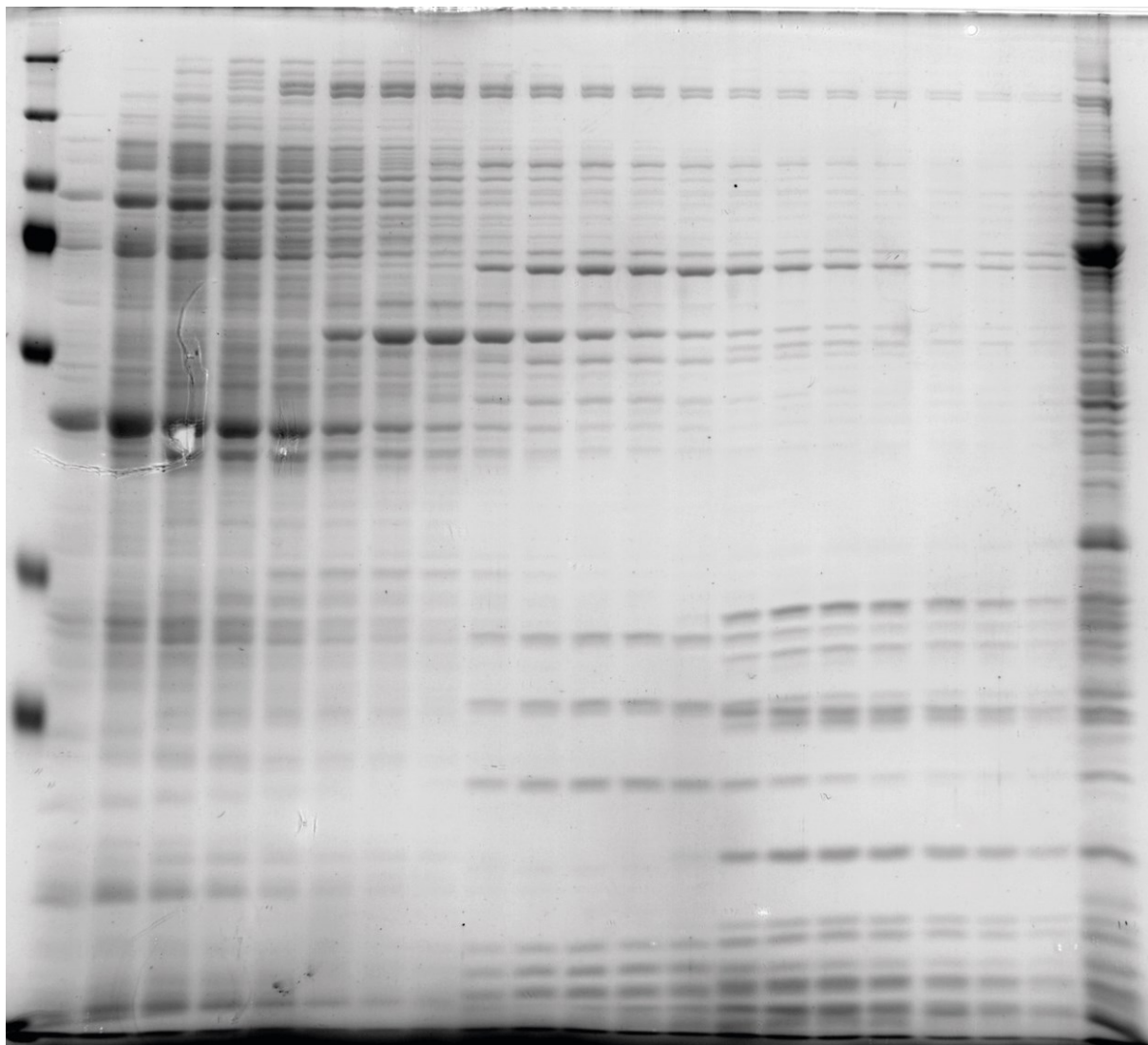

Supplement: Supplementary file 13 — Unprocessed western blots. [file 41564_2024_1616_MOESM13_ESM.pdf]

Extended Data Fig. 2

Coomassie

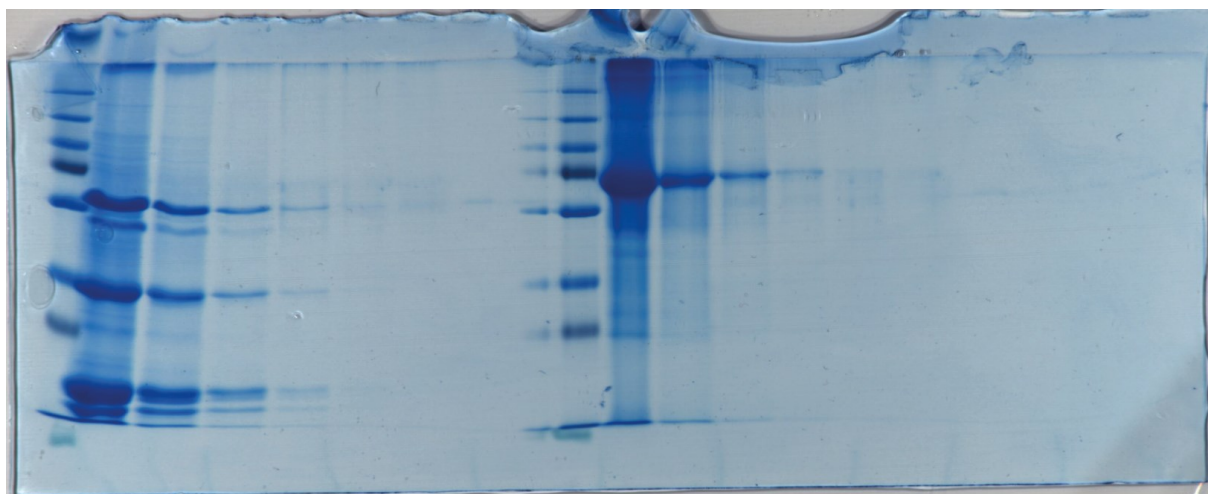

anti- $\phi$ KZ014 (1661)

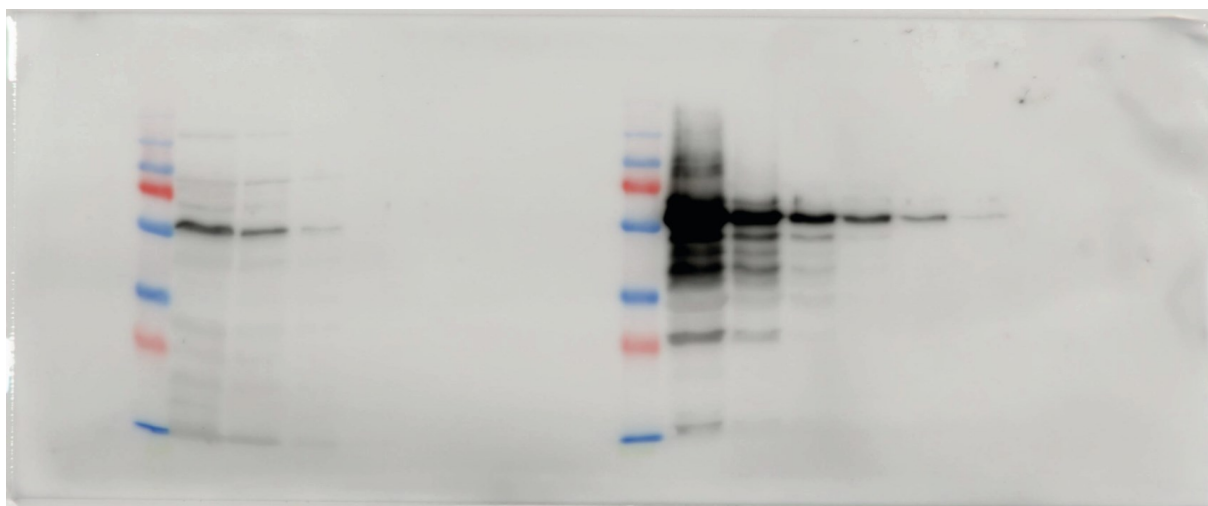

Supplement: Supplementary file 15 — Unprocessed western blots. [file 41564_2024_1616_MOESM15_ESM.pdf]

Ext. Data Fig. 3a

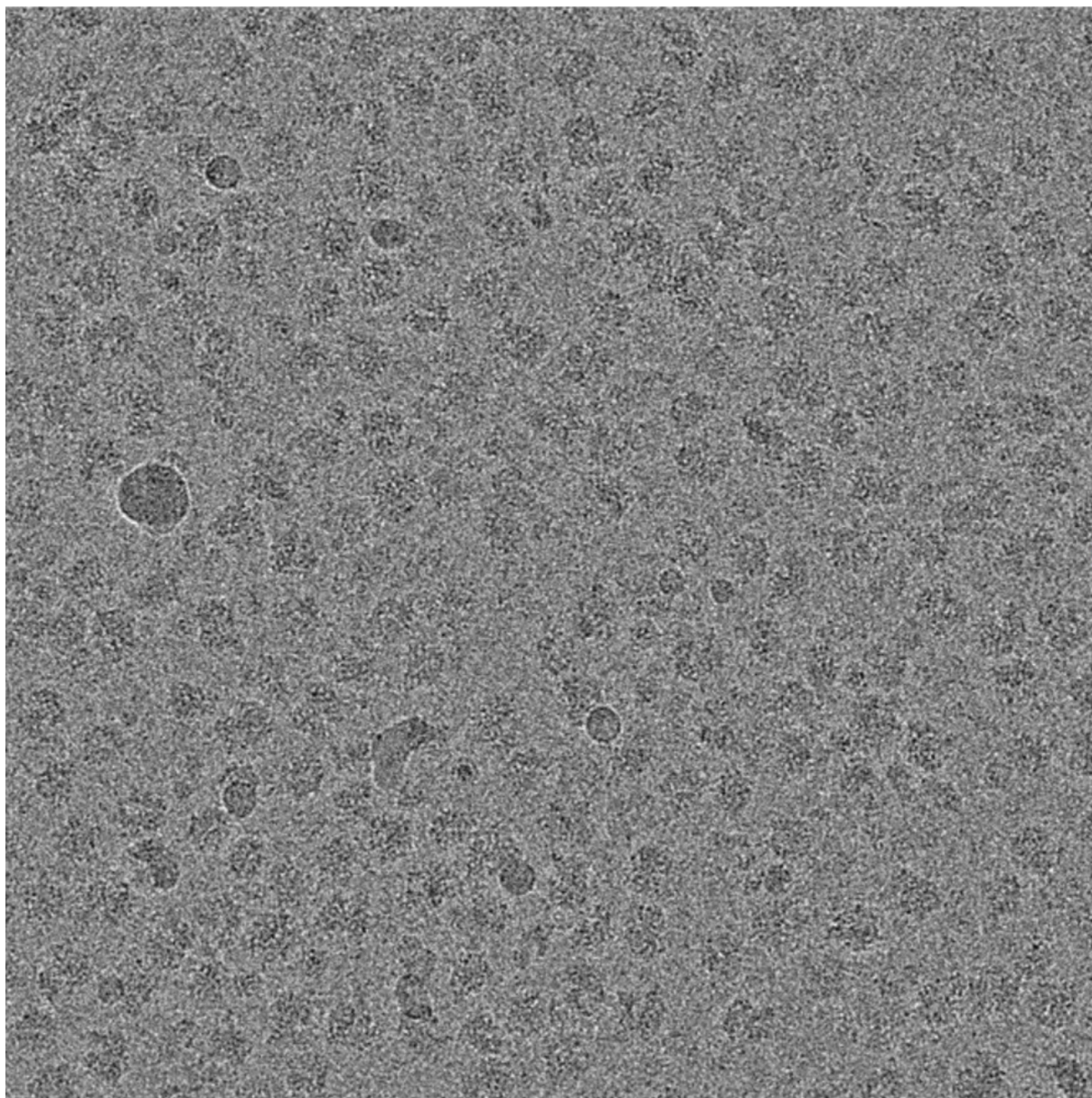

Supplement: Supplementary file 16 — Unprocessed western blots. [file 41564_2024_1616_MOESM16_ESM.pdf]
