# Supplementary material for: Phage proteins target and co-opt host ribosomes immediately upon infection
Source: Nat Microbiol. 2024 Mar 4;9(3):787–800. doi: 10.1038/s41564-024-01616-x (PMC10914614; doi:10.1038/s41564-024-01616-x)

Fig. 4b

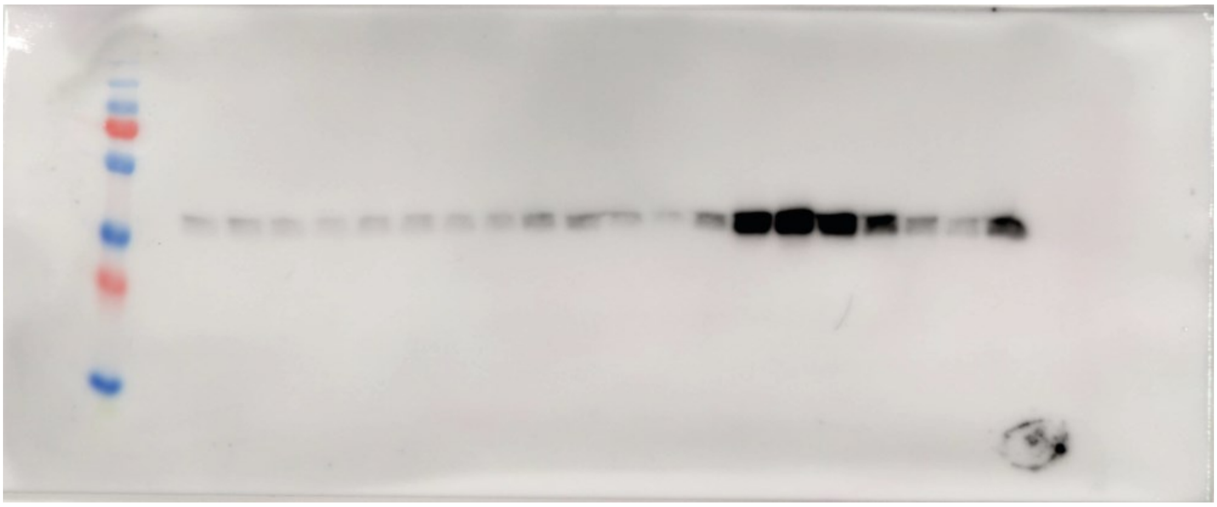

Fig. 4c

anti-ΦKZ014 (1661)

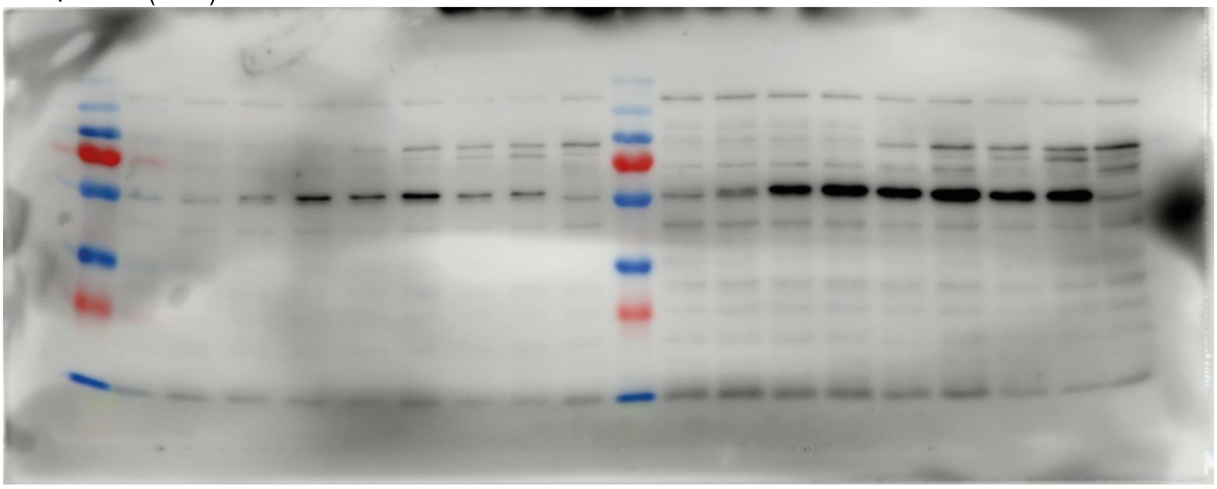

Coomassie

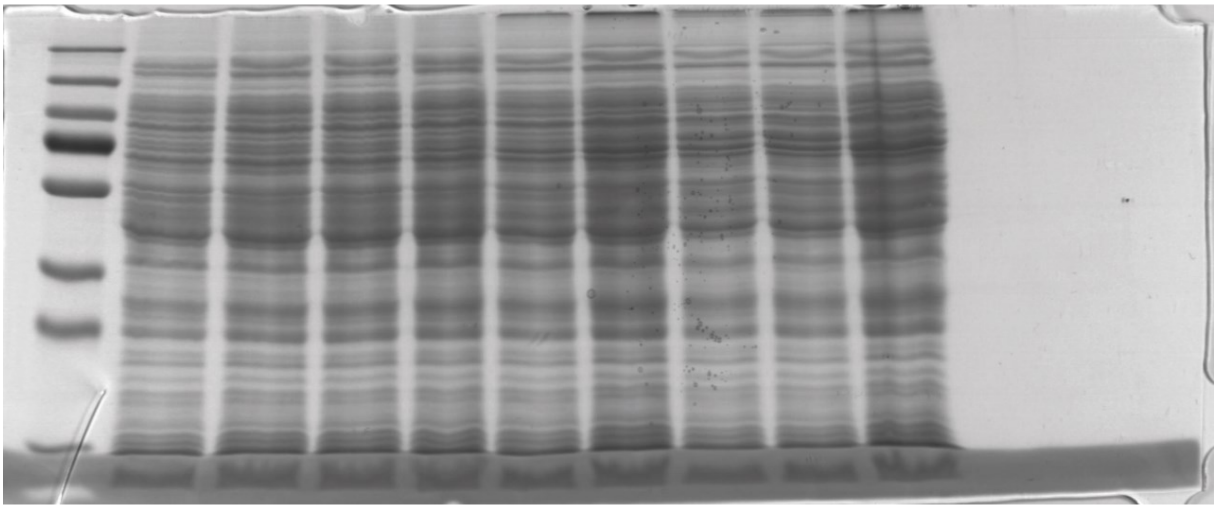

Fig. 4d

1 mM Mg

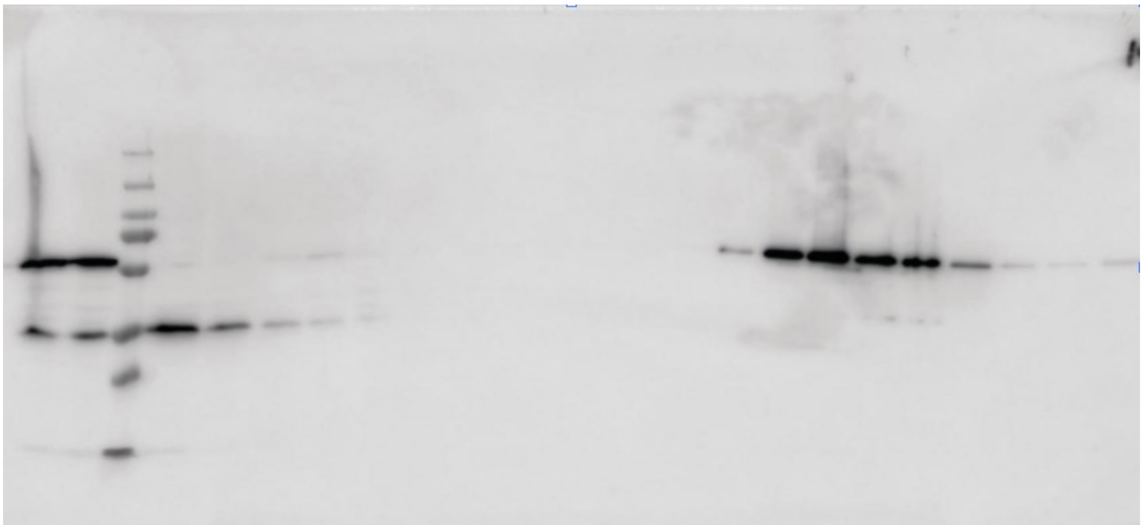

10 mM Mg

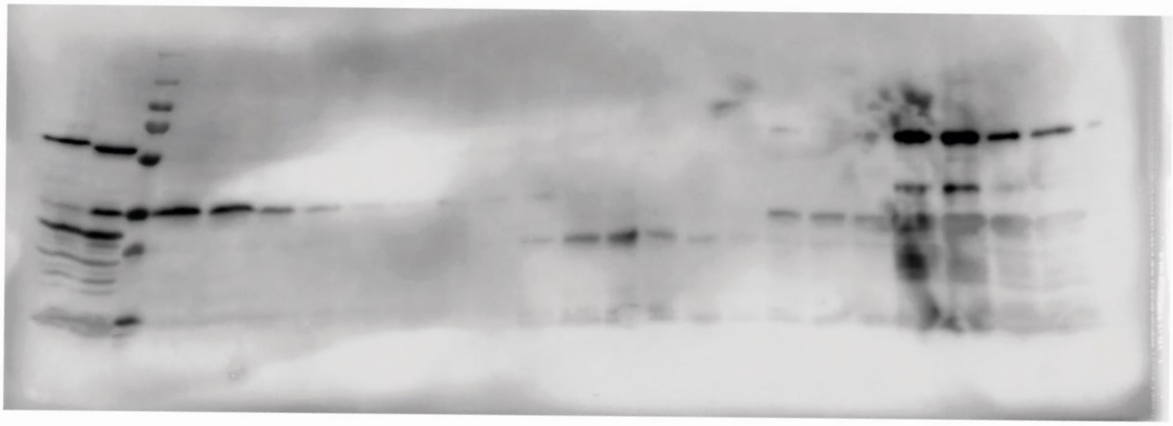

Fig. 4e

Coomassie

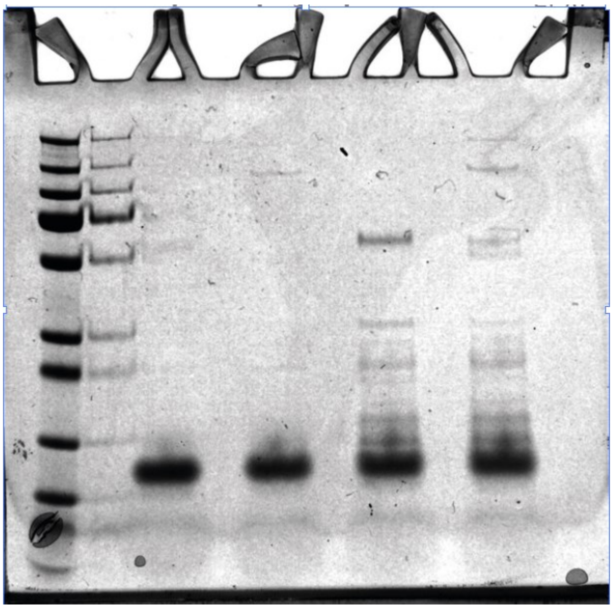

anti-FLAG

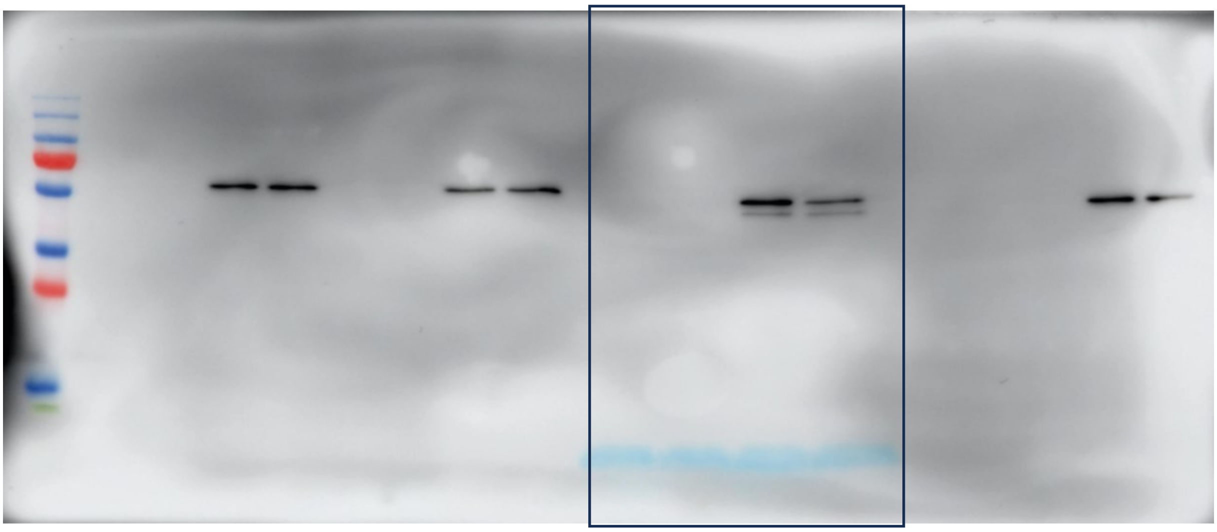

Fig. 4f

none

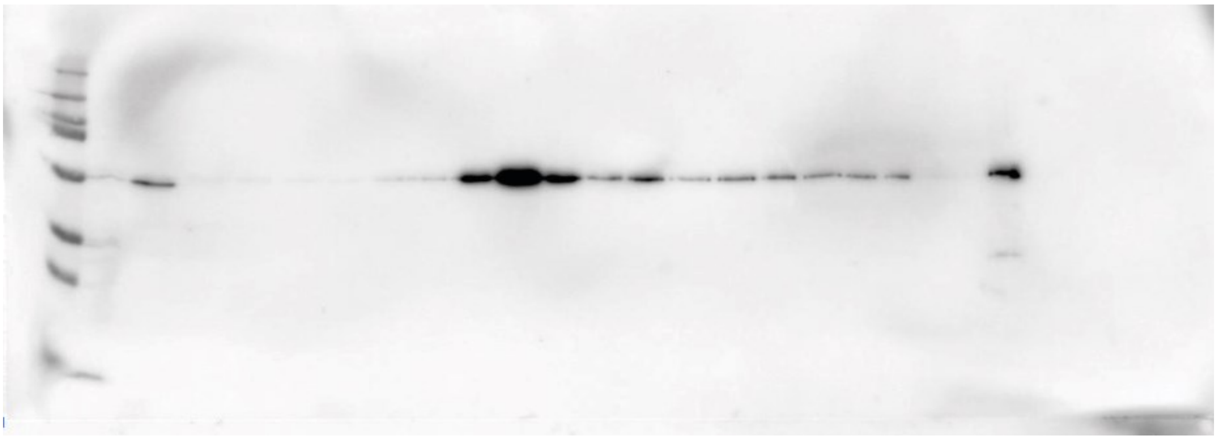

MNase

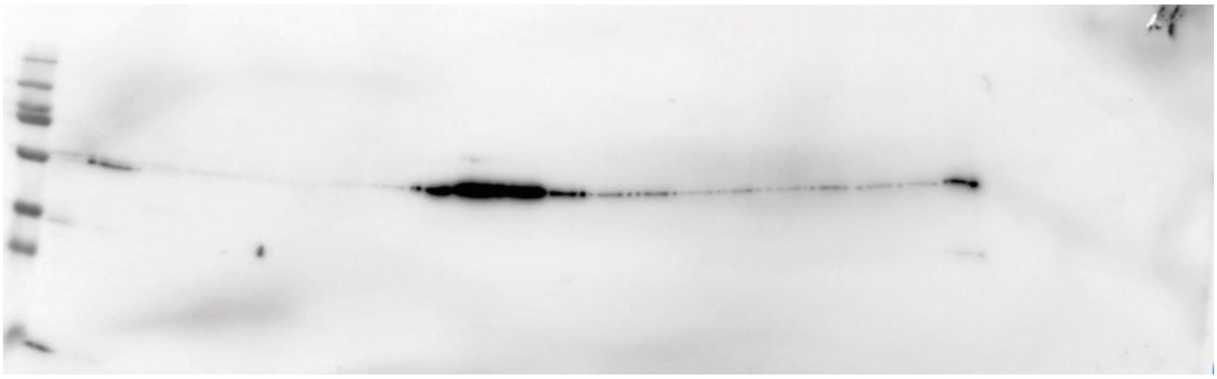

Puromycin

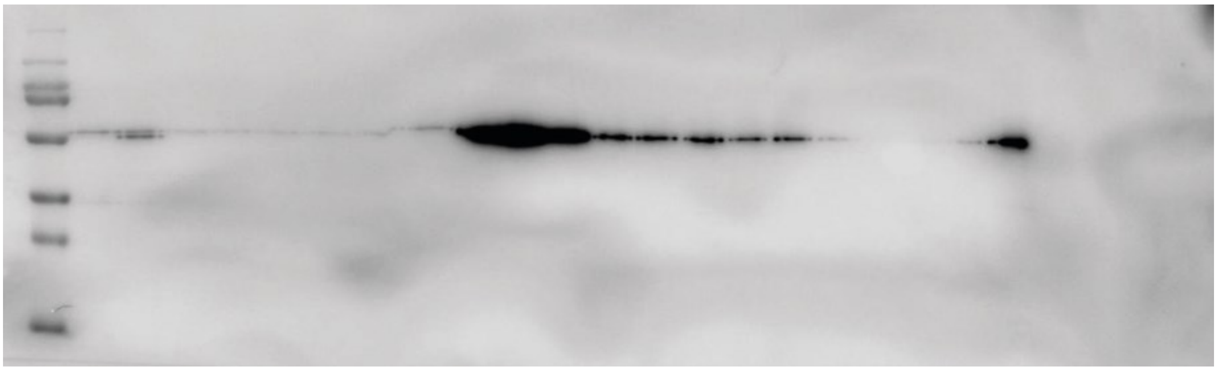

Fig. 4g

ΦKZ (wt) - anti-ΦKZ014 (1661)

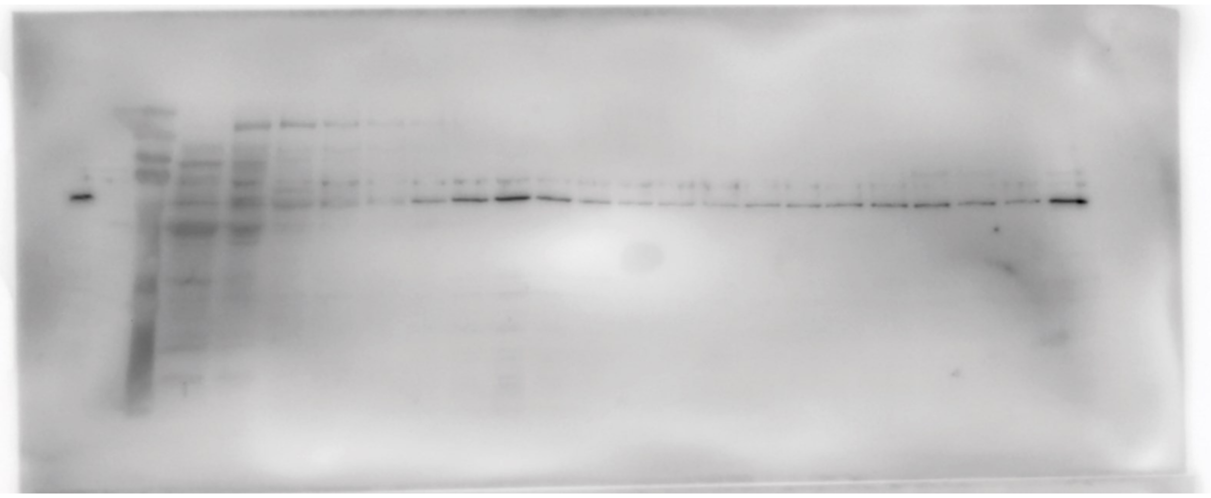

ΔΦKZ014

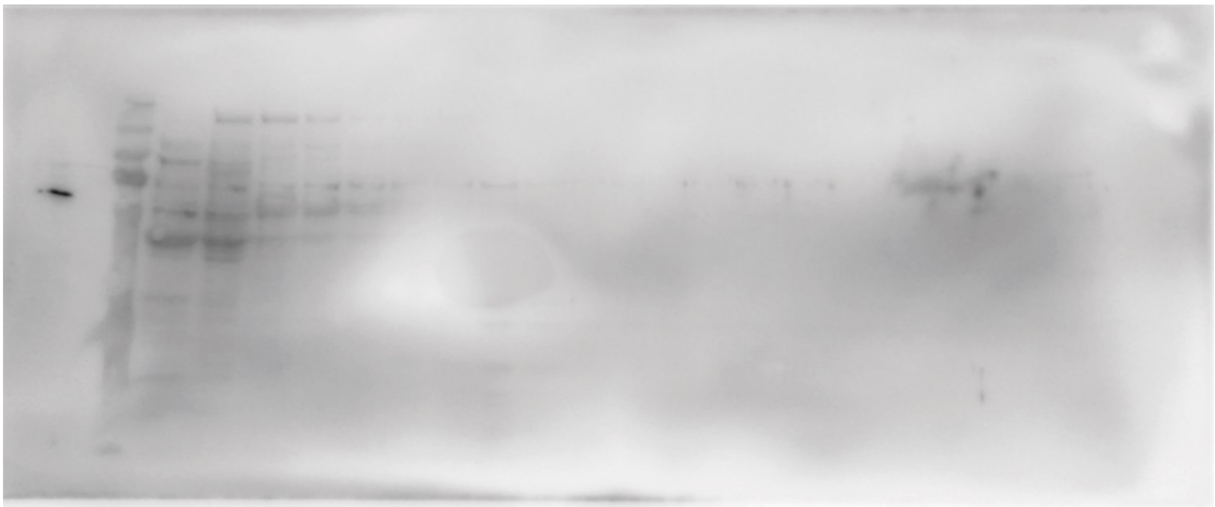

Fig. 4h

ΦKZ (wt) - anti-ΦKZ014 (1661)

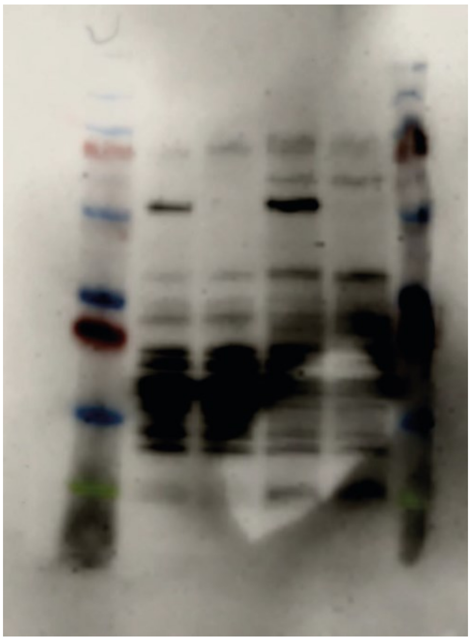

Coomassie

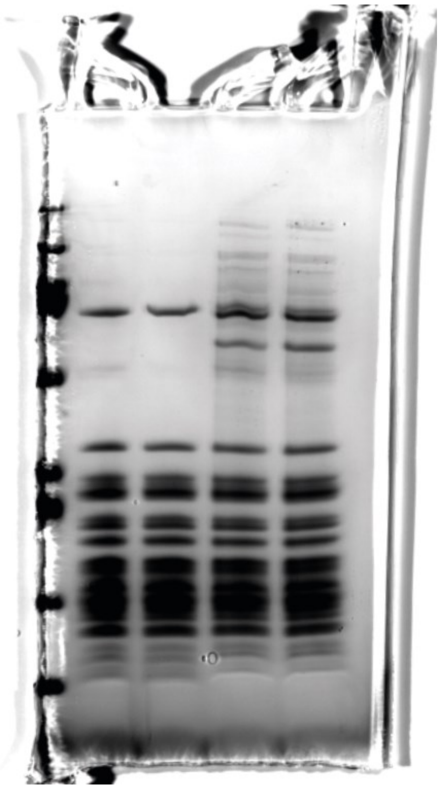

Supplement: Supplementary file 8 — Unprocessed western blots. [file 41564_2024_1616_MOESM8_ESM.pdf]
