# Supplementary material for: Phage proteins target and co-opt host ribosomes immediately upon infection
Source: Nat Microbiol. 2024 Mar 4;9(3):787–800. doi: 10.1038/s41564-024-01616-x (PMC10914614; doi:10.1038/s41564-024-01616-x)

Ext. Data Fig. 5a

Radiogram

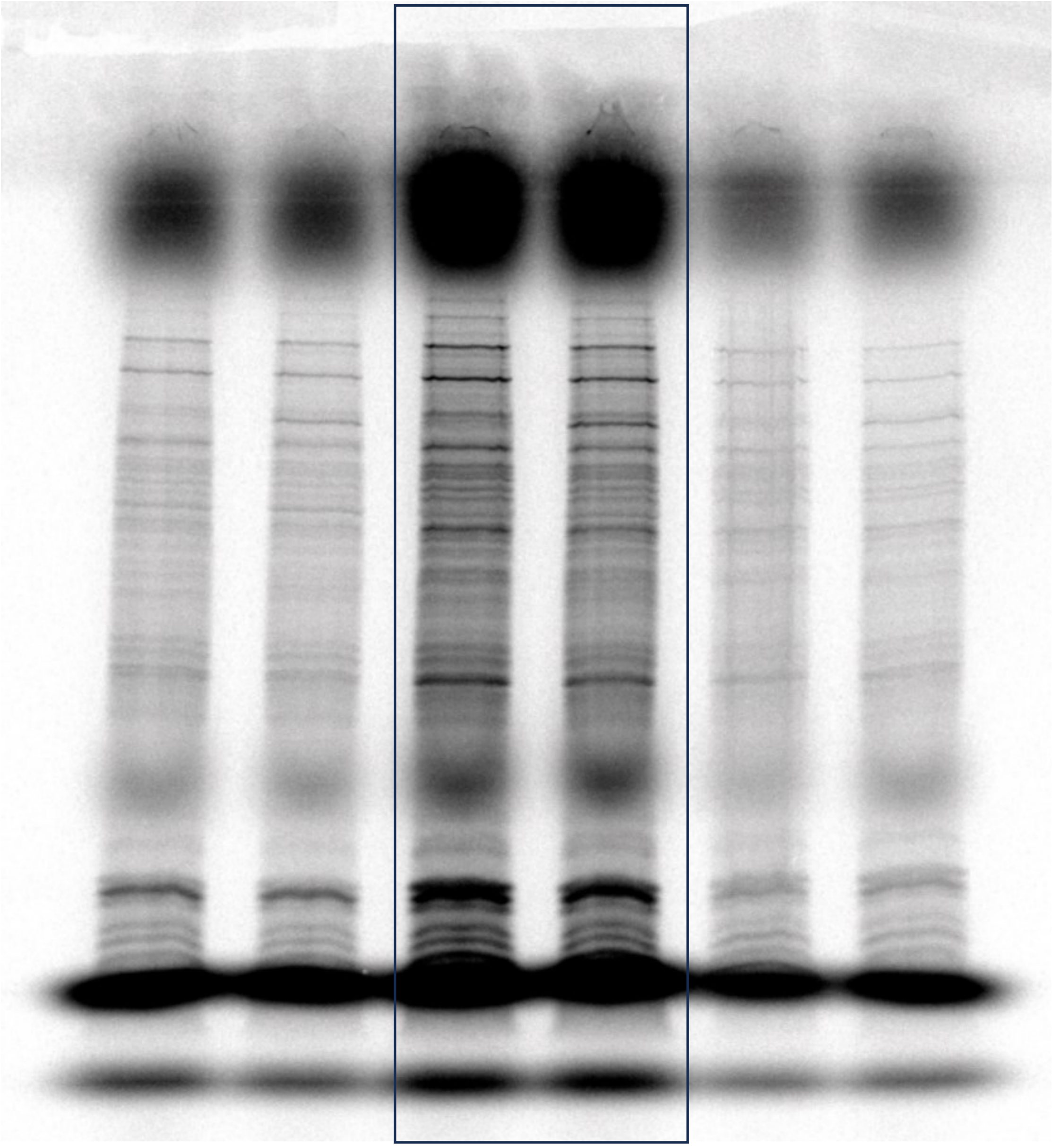

Ext. Data Fig. 5b

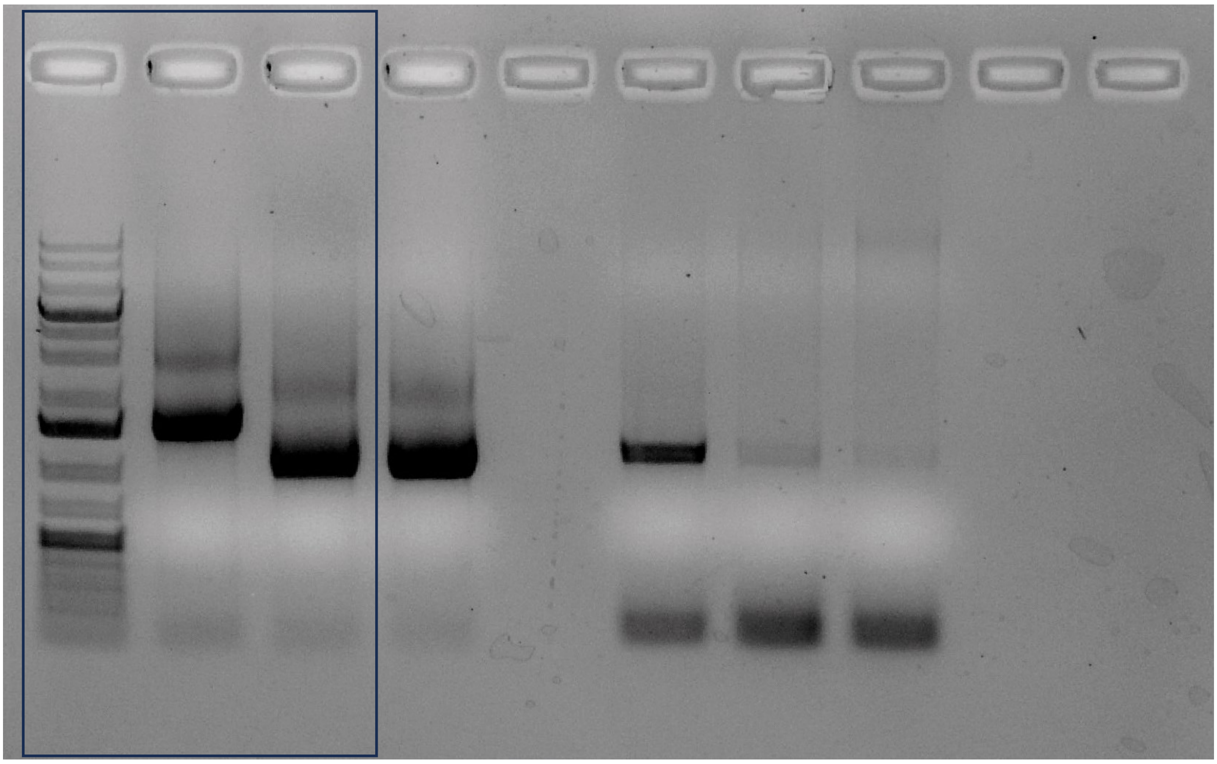

Ext. Data Fig. 5c

Infection time course, 6th lane is 25 min post infection, last lane is *ΔΦKZ014* 25 min

anti-ΦKZ014 (1661)

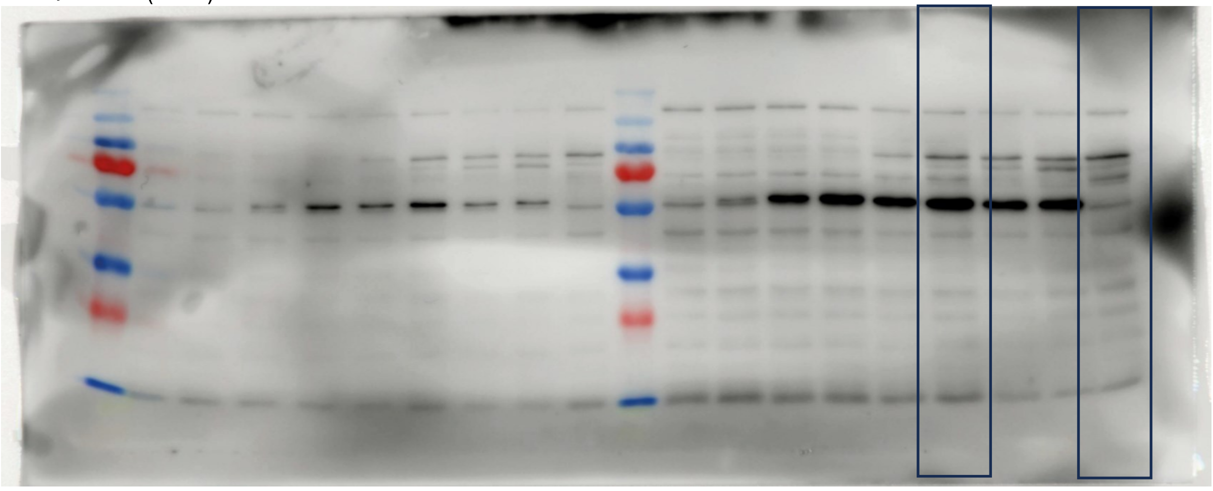

Coomassie

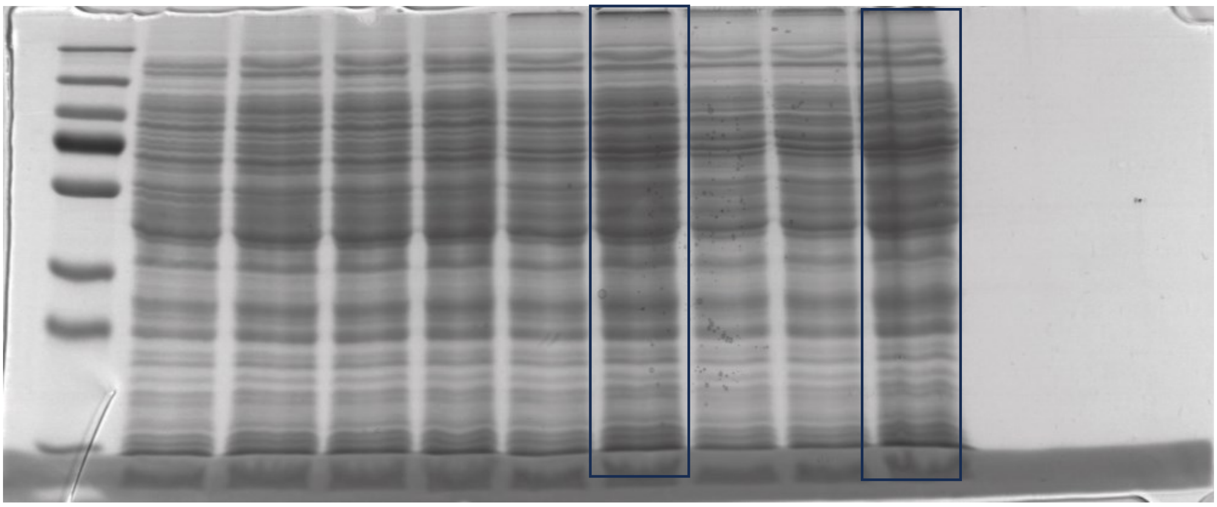

Ext. Data Fig. 5d

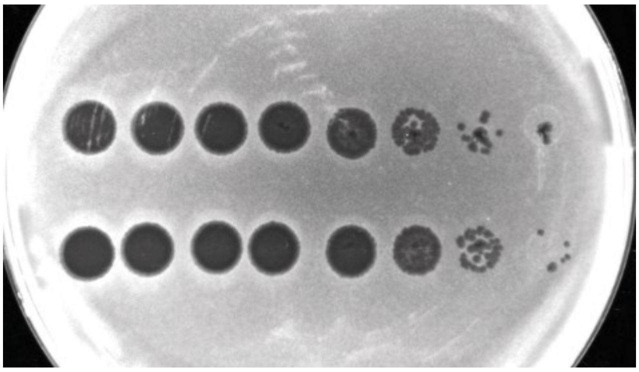

Ext. Data Fig. 5e

PAO1

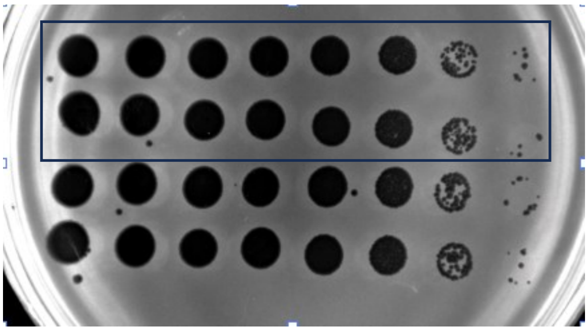

PaLo8

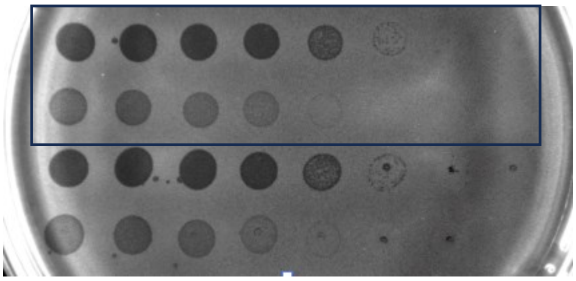

PaLo9

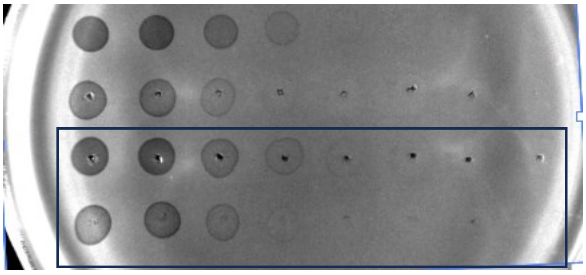

PaLo39

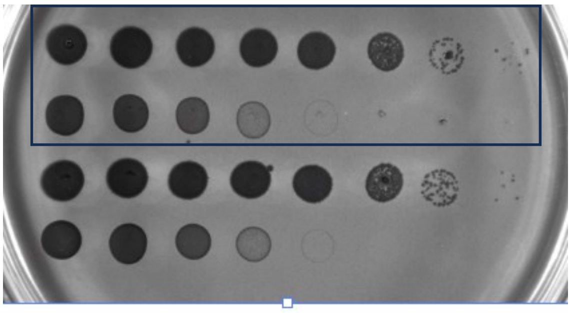

PaLo44

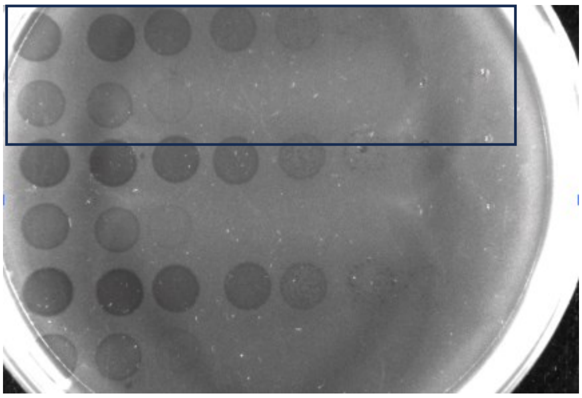

Supplement: Supplementary file 17 — Unprocessed western blots. [file 41564_2024_1616_MOESM17_ESM.pdf]
